# An objective comparison of detection and segmentation algorithms for artefacts in clinical endoscopy

**DOI:** 10.1038/s41598-020-59413-5

**Published:** 2020-02-17

**Authors:** Sharib Ali, Felix Zhou, Barbara Braden, Adam Bailey, Suhui Yang, Guanju Cheng, Pengyi Zhang, Xiaoqiong Li, Maxime Kayser, Roger D. Soberanis-Mukul, Shadi Albarqouni, Xiaokang Wang, Chunqing Wang, Seiryo Watanabe, Ilkay Oksuz, Qingtian Ning, Shufan Yang, Mohammad Azam Khan, Xiaohong W. Gao, Stefano Realdon, Maxim Loshchenov, Julia A. Schnabel, James E. East, Georges Wagnieres, Victor B. Loschenov, Enrico Grisan, Christian Daul, Walter Blondel, Jens Rittscher

**Affiliations:** 1 Institute of Biomedical Engineering, Big Data Institute, Department of Engineering Science, University of Oxford, Oxford, UK; 2 Ludwig Institute for Cancer Research, University of Oxford, Oxford, UK; 3 CRAN UMR 7039, University of Lorraine, CNRS, Nancy, France; 4 Translational Gastroenterology Unit, Nuffield Department of Medicine, Experimental Medicine Div., John Radcliffe Hospital, University of Oxford, Oxford, UK; 5 Instituto Onclologico Veneto, IOV-IRCCS, Padova, Italy; 6 Ping An Technology (Shenzhen) Co. Ltd, Shenzhen, China; 7 Beijing Institute of Technology, Beijing, China; 8 Technishe Universität München, Munich, Germany; 9 Department of Biomedical Engineering, University of California, Davis, USA; 10 Department of Bioinformatic Engineering, Osaka University, Suita, Osaka, Japan; 11 School of Biomedical Engineering and Imaging Sciences, King’s College London, London, UK; 12 Swiss Federal Institute of Technology in Lausanne (EPFL), Lausanne, Switzerland; 13 A.M. Prokhorov General Physics Institute, Russian Academy of Science, Moscow, Russia; 14 Department of Information Engineering, University of Padova, Padova, Italy; 15 Department of Ultrasound Imaging, Tiantan Hospital, Beijing, China; 16 School of Engineering, University of Glasgow, Glasgow, UK; 17 Department of Automation, Shanghai Jiao Tong University, Shanghai, China; 18 Department of Computer Science and Engineering, Korea University, Seoul, South Korea; 19 Department of Computer Science, Middlesex University, London, UK; 20 Department of Computer Engineering, Istanbul Technical University, Istanbul, Turkey; 21 School of Engineering, London South Bank University, London, UK

**Keywords:** Oesophagogastroscopy, Oesophagogastroscopy, Translational research, Translational research, Translational research

## Abstract

We present a comprehensive analysis of the submissions to the first edition of the Endoscopy Artefact Detection challenge (EAD). Using crowd-sourcing, this initiative is a step towards understanding the limitations of existing state-of-the-art computer vision methods applied to endoscopy and promoting the development of new approaches suitable for clinical translation. Endoscopy is a routine imaging technique for the detection, diagnosis and treatment of diseases in hollow-organs; the esophagus, stomach, colon, uterus and the bladder. However the nature of these organs prevent imaged tissues to be free of imaging artefacts such as bubbles, pixel saturation, organ specularity and debris, all of which pose substantial challenges for any quantitative analysis. Consequently, the potential for improved clinical outcomes through quantitative assessment of abnormal mucosal surface observed in endoscopy videos is presently not realized accurately. The EAD challenge promotes awareness of and addresses this key bottleneck problem by investigating methods that can accurately classify, localize and segment artefacts in endoscopy frames as critical prerequisite tasks. Using a diverse curated multi-institutional, multi-modality, multi-organ dataset of video frames, the accuracy and performance of 23 algorithms were objectively ranked for artefact detection and segmentation. The ability of methods to generalize to unseen datasets was also evaluated. The best performing methods (top 15%) propose deep learning strategies to reconcile variabilities in artefact appearance with respect to size, modality, occurrence and organ type. However, no single method outperformed across all tasks. Detailed analyses reveal the shortcomings of current training strategies and highlight the need for developing new optimal metrics to accurately quantify the clinical applicability of methods.

## Introduction

Endoscopy is a routine clinical procedure used for the detection, follow-up and treatment of disease such as cancer and inflammation in hollow organs and body cavities; ear, nose, throat, urinary tract, oesophagus, stomach and colon, which otherwise would be difficult to examine. During the endoscopic procedure an *endoscope*, a long, thin, rigid or flexible tube with a light source and a camera at its tip is inserted. In the clinical setting, endoscopes are typically connected to an external monitor for visualization. With the help of this display, the trained clinical endoscopist navigates within an organ, conducts reporting, acquires biopsies, and if necessary performs minimally invasive surgery. However, these processes are highly dependent upon the operator’s experience and navigation skills. Despite recent hardware improvements of clinical endoscopes allowing high definition and high frame rate image capture, the quality of endoscopic videos is still compromised. This is mostly due to non-optimal reflection of light, unavoidable tissue movements, large differences in organ shape and surface texture as well as occlusions caused by bodily fluids and debris. Most common imaging artefacts include the over- and under-exposure of image regions due to changes in illumination and organ topology (termed “saturation” or “contrast”, respectively), blur due to unsteady hand motion of endoscopists and local organ motion, and specularity due to light reflection from smooth organ surfaces. The presence of fluids and bubbles also influences the visual interpretation of the mucosal surface^1^. Often, more than 60% of an endoscopy video frame and nearly 70% of an endoscopy video sequence^1^ can be corrupted by a multitude of artefacts (see also Suppl. Fig. 1). These imaging artefacts not only present difficulty in visualizing the underlying tissues, but also severely impede quantitative analysis. Automated analyses of underlying pathologies often fail and lead to errorneous detections. Many sophisticated methods in literature used for identifying lesions such as *polyp detection* in bowel disease suffer from loss in accuracy due to the presence of image artefacts^2^. Tiny erosions in colitis are hard to detect and the presence of specularities or saturated pixels make this task even more challenging for both human specialists and computer guided methods. Furthermore, methods such as automatic video frame retrieval for reporting^3^, and 2D video mosaicking^4^ or 3D surface reconstruction^5^ for producing extended panoramic images for disease monitoring and surgical planning require continuous video and are severely compromised by corrupted frames. Thus, it is undoubtedly necessary and imperative to identify and localize artefacts so that adequate video frame quality restoration can be applied before building any computer assisted clinical analysis techniques. Accurate artefact detection is therefore a critical bottleneck that must be first resolved to pave the way forward towards building any computer-aided endoscopy tools. In addition, artefact detection and its quantification can provide a measure of the endoscopic procedure quality.

Universal endoscopic artefact detection is highly challenging. This is due to (i) large variation in tissue appearance under different endoscopy modalities used to aid endoscopic inspection such as white light, fluorescent and narrow band imaging (Supplementary Note 1), (ii) the presence of diverse image artefacts mostly caused by different physical phenomena (Suppl. Figs. 2, 3), (iii) the large variability in each individual artefact type with respect to their size, location and appearance, (iv) the frequent colocalization and overlap of small and large area artefacts of different nature (Suppl. Figs. 3c,d, 4), and (v) the lack of distinctive image features that distinguish and define each artefact. Bubbles are an exemplary example of the latter problem. Within a bubble, the underlying tissue is still visible (albeit possibly optically distorted) and its edges can span across a small or large area. Endoscopists characterize bubbles by the presence of a “protrusion” from the surface and the presence of specularity, global and local features which cannot easily be modelled numerically. An alternative to modelling is to *learn* the appearance statistics of artefacts using labelled images. Unfortunately, unlike natural images^6,7^ that can be readily crowd-sourced, labelled biomedical images are notoriously scarce and difficult to annotate. The ethical requirements and subtler image features of medical images require annotators with significant domain expertise. Current literature attempt to circumvent this data limitation by adapting methods pretrained on large natural image datasets. However, this fails due to the inability of these methods to generalize on large endoscopy data samples. This is because the evaluated image datasets are often collected in-house with limited image diversity that do not fully reflect the tissue appearance and different image acquisition practices worldwide. Most works have been performed on short video clips, selected artefact type, single imaging modality and single organ datasets^8–11^.

We initiated the Endoscopy Artefact Detection Challenge (EAD2019) to enable a more comprehensive and objective benchmarking of endoscopic imaging artefact detection and segmentation algorithms by creating a diverse repository of annotated endoscopy video frames^12,13^ (Suppl. Fig. 2). EAD is a crowd-sourcing initiative that challenges researchers and computational experts to test and build their algorithms on a common benchmark clinical endoscopy dataset. To best capture the image variation in endoscopy, the assembled image frames were sourced from multiple organs, imaging modalities, endoscope manufacturers, and patients of different ethnic backgrounds (Suppl. Fig. 3a). Altogether the dataset captures seven prevalent artefact classes as identified by expert endoscopists^13^ (Suppl. Fig. 3b). The impact of posing the complex problem of artefact detection in diverse clinical endoscopy data through a challenge can be seen in twofold: (1) to communicate with the wider community to best address the most fundamental limitation that impedes quantification of endoscopy data worldwide, and (2) to deliver improved patient monitoring with the endoscopy procedure by image artefact quantification and the development of enhanced computer-aided endoscopy tools.

In the EAD2019 competition, the developed methods from participants were evaluated on a web-based platform with standard computer vision metrics. In this paper, we present an in-depth analysis of the EAD results including the performance impact due to the dataset size and artefact types. We aim to highlight the current best approaches for handling such a diverse endoscopy imaging dataset and to direct developers and researchers to open challenges that we believe are still insufficiently addressed by current algorithms. The drawbacks of standard evaluation metrics to highlight the strength and weakness of individual methods are also discussed. We additionally introduce a holistic analysis of individual algorithms to identify and measure the clinical applicability of the developed methods in this competition. Importantly, the EAD challenge is an open-source initiative that will continue to remain open online for submission (https://ead2019.grand-challenge.org/). Most methods submitted to the EAD2019 workshop are publicly available through the challenge website.

## Materials and Challenge Tasks

### Dataset

The EAD2019 dataset is the first publically available dataset aiming to capture the wide visual diversity in endoscopic videos acquired in everyday clinical settings (Suppl. Fig. 2). Supplementary Note I and Suppl. Figs. 3, 4 provides a detailed breakdown of the dataset and its construction. Briefly, the EAD 2019 dataset identifies seven prevalent image artefact types or classes: (1) specularity, (2) saturation, (3) artefact, (4) blur, (5) contrast, (6) bubble and (7) instrument^12^, and is multi-organ, multi-modal, multi-patient and multi-ethnic, (Suppl. Fig. 3a). The dataset contains images from multiple patients of different ethnic origins. The defined class types aim to capture the most prevalent artefact types worldwide. The training dataset for detection and out-of-sample generalization tasks contain 2192 unique video frames with bounding box annotations and class labels from all 7 classes. A subset collection of 475 video frames additionally have binary image mask annotations for 5 classes (excluding blur and contrast) for the semantic segmentation task. The test dataset with reference annotations (unavailable to the public) contain an additional 195, 122 and 51 video frames for detection, segmentation and out-of-sample generalization tasks, respectively. To capture the natural frequency and inherent multi-class features of endoscopic artefacts, we allow labelling of artefacts in the same spatial location with multiple relevant class labels. This is unlike natural images^6^ or any other endoscopic dataset aimed at finding disease^14^ where class labels are considered mutually exclusive. The frames presented in this dataset were extracted from nearly 125 endoscopic videos provided by collaborating institutions. The data for detection and segmentation sub-challenges were collected from five international institutions: John Radcliffe Hospital, Oxford, UK; ICL Cancer Institute, Nancy, France; Ambroise Paré Hospital of Boulogne-Billancourt, Paris, France; University Hospital Vaudois, Lausanne, Switzerland and the Botkin Clinical City Hospital, Moscow, Russia. Frames for out-of-sample generalization come provided by a sixth institute, Instituto Oncologico Veneto, Padova, Italy. During the challenge, the training datasets were released in two separate batches. The first batch provided the annotations for the detection sub-challenge, while the second batch supplemented the detection sub-challenge and provided the annotations for the segmentation sub-challenge. By staggering the release of the detection training dataset, participants were able to use either the first or second batch to test detection out-of-sample generalization. Participants had a total of three months to complete their submissions. The test data (excluding the reference annotations) were kept secret and only provided one month prior to the final workshop conference. An online system evaluated of participants results with respect to (hidden) ground truth labels.

The EAD2019 training dataset can be downloaded at the open access Mendeley data repository. A comprehensive companion open-source software to assist users in data preparation and evaluation of predictions.

### Challenge tasks

**Figure 1:**
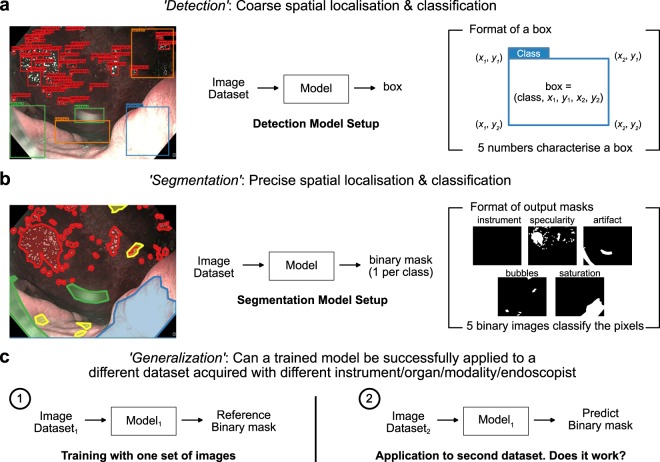
The three sub-challenges of the Endoscopy Artefact Detection (EAD) challenge. (**a**) The “detection” task is aimed at coarse localization and classification of each image artefact. Given an input image (left) a detection model (middle) outputs the artefact class and coordinates of the containing bounding box defined by the top left (*x*_1_, *y*_1_) and bottom right corners (*x*_2_, *y*_2_) of the box (right). (**b**) The “segmentation” task is aimed at finer spatial localization through the precise delineation of artefact boundaries. Given an input image (left), a segmentation model outputs binary images (right) denoting the presence (‘1’) or absence (‘0’) of each artefact class. (**c**) The “out-of-sample generalization” task is aimed at assessing the ability of a model (model_1_) trained on one dataset (dataset_1_) model_1_ (left) to detect artefacts in an unseen dataset (dataset_2_) comprising the same set of class labels but with different data attributes such as data modalities or instrument or acquisition center or a combination of factors.

To enable detailed assessment of algorithm performance, the overall artefact detection and classification problem was subdivided into three sub-challenges: detection, segmentation and out-of-sample generalization (Fig. 1 and Suppl. Fig. 1). Detection targets the coarse localization of image artefacts, identification of their class type and spatial location (given by the top-left and bottom-right coordinates of a rectangular bounding box in Fig. 1a). Segmentation addresses pixel-wise localization of each artefact class giving the exact shape of artefacts within a class. Finally, out-of-sample generalization assesses the ability to apply a model trained on a given dataset “1” to a completely different subset of dataset “2” comprising similar images but from different data source (e.g., different manufacturer, different organ etc.).

## Participants, Algorithms and Submission Handling

29 teams from 9 countries and 4 continents participated in EAD challenge 2019, all providing results for at least one of the 3 sub-challenges. 23 participants submitted detection, 16 participants segmentation and 19 participants submitted out-of-sample generalization results. Detection and out-of-sample generalization submissions were received by the EAD organizers as text files giving the predicted class, confidence and coordinates of the predicted bounding box (Fig. 1a). Submissions to the segmentation task were received as binary segmentation masks, one for each class (Fig. 1b). Participants were also asked to submit a technical abstract (2–4 pages) which was independently peer-reviewed by at least two computer vision scientists. Successful papers were compiled into an online proceeding(http://ceur-ws.org/Vol-2366). Table 1 summarises the technical details of the submitted solutions for detection, Table 2 and Table 3 presents their comparative performance evaluation while Table 4 summarises and compares the segmentation solutions. Interestingly no dominant architecture or approach arose. The most popular detection solutions were based on Mask R-CNN^15^, RetinaNet^16^ and Cascade R-CNN^17^ and the most popular segmentation solutions were Mask R-CNN^15^ and DeepLabV3^18,19^. See Supplementary Note IV for a condensed description of methods used by the top 30% participants.

**Table 1:** Summary of participant algorithms for multi-class artefact detection and out-of-sample generalization.

Team	Algorithm	Prep.	Nature	Basis-ofchoice	Backbone	Data Aug.	Pretrained	Computation		
								GPU	Train time, *hr*	Test time, *s*
yangsuhui	Cascade R-CNN +FPN	Norm. (0–1)	Ensemble	Accuracy	ResNet101	Yes	None	1080 Ti	47	57
zhangPY	Mask-aided RCNN	Norm. (0–1)	Symbiosis	Context	ResNet50, ResNet101 +FPNResNet50, ResNet50 +FPNResNet50	Yes	None	1080 Ti	47	57
Keisecker	RetinaNet	Norm. (0-1)	Ensemble	Accuracy	ResNet50, ResNet-101, ResNet-152	Yes	COCO, ImageNet1k	K80	8	0.19
michaelqiyao	Cascade R-CNN	Norm. (0-1)	Cascading	Accuracy	ResNet101	Yes	COCO	—	—	—
ilkayoksuz	RetinaNet	-	Focal loss	Accuracy speed	ResNet-152	Yes	COCO	K80	26	2.00
swtnb	DNN +Mask R-CNN +YOLOv3	Patch	Symbiosis	Context	ResNet-101	Yes	COCO	—	—	—
akhanss	RetinaNet	Norm.	Focal loss	Accuracy speed	ResNet-101	Yes	ImageNet	TITAN XP	3	1.00
XiaokangWang	Faster RCNN	Patch, scaling	Feature pyramid	Context	FPN-ResNet50	Yes	COCO	1x1070	3.5	2.40
nqt52798669	Cascade RCNN	Patch	Cascading	Accuracy	ResNet-101, DLA-60	No	None	2x1080Ti	—	—
ShufanYang	Unet-D	Bg. subs.	Semantic	Context speed	ResNet-50	No	P. VOC 12	1x1080Ti	12	0.04

Most methods also used non-maximum suppression (NMS, Supplementary Note III) for post-processing to obtain the final bounding box predictions. Teams are ordered according to their ranking on the online leaderboard (Table 2). “—” denotes unavailable information.

**Table 2:** Team scores for artefact class detection and out-of-sample generalization.

Team name	Detection			Generalization		
	mAP_d_	IoU_d_	score_d_	mAP_g_	IoU_g_	dev_g_
yangsuhui	**0.3235**	**0.4172**	**0.361**	**0.3187**	0.0734	0.1018
ZhangPY	0.3117	**0.4051**	**0.3491**	**0.3518**	0.0889	**0.0984**
Keisecker	0.3087	**0.3997**	**0.3451**	0.2848	**0.3902**	**0.0696**
VegZhang	**0.3371**	**0.3517**	**0.3429**	**0.3991**	0.1783	0.101
YWa	**0.3842**	0.2368	**0.3252**	**0.3746**	0.1481	**0.0424**
michaelqiyao	**0.3842**	0.2368	**0.3252**	**0.3746**	0.1780	**0.0742**
ilkayoksuz	0.2719	0.3456	0.3014	0.2974	0.0688	0.0859
swtnb	0.2901	0.318	0.3013	0.2914	**0.2547**	0.0854
Witt	**0.3148**	0.2621	0.2937	0.2897	0.1854	0.1003
akhanss	0.2581	0.333	0.288	0.2187	**0.2262**	0.0770
XiaokangWang	0.2621	0.3205	0.2855	0.2515	0.2058	0.0728
a545306097	0.2547	0.2719	0.2616	0.1122	**0.2244**	0.1298
nqt52798669	0.3068	0.1222	0.233	0.3154	0.0871	**0.0515**
ShufanYang	0.2208	0.1955	0.2107	0.1931	0.1365	0.0478
xiaohong1	0.2416	**0.3482**	0.2842	0.1764	**0.2671**	0.0555
Faster R-CNN (baseline)	0.2226	0.2751	0.2436	0.2172	0.1647	0.0893
Retinanet (baseline)	0.2135	0.2270	0.2189	0.2499	0.1679	0.0665
Merged (super baseline)	0.3331	0.3793	0.3516	0.3433	0.2610	0.0610

Off-the-shelf Faster R-CNN [20] and RetinaNet^16^ are reported as baselines (as labeled) for comparison. We also include the performance of a super classifier denoted ‘Merged’ constructed from merging the predicted bounding boxes of all participants. Performance evaluated using the detection or out-of-sample generalization dataset is differentiated by subscripts ‘d’ and ‘g’, respectively. Teams are ordered in decreasing order of score_d_. The better the method, the higher the mAP and IoU, the lower the dev_g_. Top 5 values for each evaluation metric is shown in bold.

**Table 3:** Class specific mAP and IoU scores for artefact detection for top 30% participants.

Team name	Class specific detection													
	Blur		Contrast		Specularity		Saturation		IA		Bubbles		Instrument	
	mAP	IoU	mAP	IoU	mAP	IoU	mAP	IoU	mAP	IoU	mAP	IoU	mAP	IoU
yangsuhui	0.28	0.45	0.44	0.29	**0.48**	0.30	0.48	0.33	**0.32**	0.32	0.06	0.77*	0.26	0.46
ZhangPY	0.33	0.41	0.41	**0.41**	0.35	**0.34**	0.45	0.38	0.20	**0.40**	0.20	0.27	0.24	**0.62**
Keisecker	0.31	**0.50**	0.40	0.38	0.36	0.29	0.38	**0.43**	0.23	0.37	0.18	0.26	**0.30**	0.56
michaelqiyao	**0.37**	0.22	**0.47**	0.25	**0.48**	0.22	**0.52**	0.29	0.31	0.26	**0.24**	0.08	**0.30**	0.33
ilkayoksuz	0.25	0.33	0.32	0.34	0.27	0.30	0.35	0.36	0.24	0.38	0.19	0.25	0.29	0.45
swtnb	0.34	0.23	0.44	0.21	0.28	0.27	0.32	0.36	0.23	0.33	0.17	**0.30**	0.25	0.52
Faster R-CNN	0.17	0.35	0.33	0.21	0.21	0.37	0.33	0.15	0.15	0.19	0.11	0.10	0.21	0.45
Retinanet	0.21	0.20	0.32	0.25	0.12	0.17	0.39	0.32	0.12	0.24	0.18	0.15	0.16	0.27
Merged	0.32	0.37	0.45	0.37	0.37	0.31	0.43	0.41	0.26	0.39	0.23	0.30	0.27	0.51

Off-the-shelf Faster R-CNN^20^, RetinaNet^16^ and a super detector, ‘Merged’ constructed by merging all consensus detections among participants are reported as baselines for comparison. Teams are presented in decreasing order of detection score, (score_d_). The better the method, the higher the mAP and IoU.

**Table 4:** Methods and team scores for the semantic segmentation of artefacts.

Team	Method	Nature	Backbone	Evaluation metric						
				DSC	Jaccard	Overlap	F2-score	PPV	Recall	s-score
yangsuhui	DeepLabV3+	Ensemble	ResNet-101 + MobileNetv2	**0.6810**	**0.6416**	**0.6612**	**0.6779**	0.8789	0.7148	**0.6654**
swtnb	Mask R-CNN+YOLOv3	Symbiosis	ResNet-101	0.6496	0.6041	0.6269	0.6585	0.7515	**0.7594**	0.6348
YWa	—	—	—	0.6392	0.6021	0.6206	0.6243	**0.9039**	0.6602	0.6216
VegZhang	—	—	—	0.6141	0.5831	0.6185	0.6185	0.8386	0.6839	0.6036
michaelqiyao	PSPNet	Pyramid pooling	ResNet-34	0.6141	0.5787	0.5964	0.6171	0.8164	0.6987	0.6016
Ig920810	—	—	—	0.6079	0.5684	0.5882	0.5972	0.8189	0.6802	0.5904
Weiminson	—	—	—	0.6011	0.5631	0.5821	0.5839	0.8375	0.6598	0.5825
ZhangPY	Mask-aided R-CNN	Symbiosis	ResNet-101	0.5719	0.5397	0.5558	0.5701	0.7719	0.6581	0.5594
nqt52798669	Cascaded R-CNN +DLA	Ensemble	ResNet-101 + DLA60	0.5414	0.4998	0.506	0.5331	0.6290	0.6887	0.5237
ShuganYang	U-Net-D	Semantic	ResNet-50	0.4119	0.3797	0.3958	0.3998	0.6407	0.6360	0.3968
Baseline	U-Net	Semantic	FCN	0.5490	0.5030	0.5260	0.5580	0.6691	0.7488	0.5340
Super Baseline	Merged	Semantic	—	0.6782	0.6356	0.6569	0.6703	0.8747	0.7178	0.6603

Teams are ordered by decreasing s-score. Off-the-shelf U-Net^24^ is reported as a baseline (as labeled) for comparison. We also include the performance of a super segmentation denoted “Merged” constructed by keeping the consensus predicted segmentations from all teams. The most popular architectures were variations of the popular two-stage Mask R-CNN^15^ network (*swtnb, ZhangPY*). The deep encoder-decoder DeepLabV3 + ^19^ of *yangsuhui* obtained the highest s-score. However, *YWa* scored the highest values for PPV. ‘—’ denotes missing information.

## Performance Evaluation Criteria

We give a brief overview of evaluation criteria. Detailed descriptions are given in Supplementary Note II.

### Evaluation criteria for detection

#### Intersection over union (IoU) and Jaccard index (J)

A measure that quantifies the area overlap between two spatial regions using the intersection-over-union between reference or ground-truth (denoted *R*) and predicted bounding boxes and segmentations (denoted *S*), ${\rm{IoU}}\,(R,S)\,{\rm{or}}\,{\rm{J}}\,(R,S)\,=\,\frac{|R\cap S|}{|R\cup S|}$ where |·| denote the set cardinality (Suppl. Fig. 5a). The IoU is 0 for no overlap and 1 for perfect overlap. In the context of image segmentation the IoU is also referred to as the Jaccard index.

#### Mean average precision (mAP)

Measures how well a detection method is able to retrieve all reference boxes when predictions are ranked by decreasing confidence. We define a positive “match” between a reference and predicted box if IoU ≥0.25. The mAP ranges between 0 for no retrieval and 1 for perfect retrieval. The higher the mAP the better the detector performance. See Supplementary Note II, Suppl. Fig. 5 for the technical details and how the number of positive matches differ for alternative IoU thresholds.

#### Detection score

Participants were ranked on a final weighted mean score (score_d_) = 0.6 mAP + 0.4 IoU to favour retrieval of all artefacts.

#### mAP-IoU ratio check for valid submissions

A check was placed on mAP and IoU to discourage the participants from artificially increasing the detection score through biasing mAP and IoU using early or late stopping during training. The mean IoU of valid submissions was constrained to be ± 30% of the mean mAP that is 0.7 < IoU/mAP < 1.3.

### Evaluation criteria for segmentation

#### Dice similarity coefficient (DSC)

A spatial overlap measure for segmentation similar to IoU defined as ${\rm{DSC}}\,(R,S)\,=\,\frac{2|R\,\cap \,S|}{|R|+|S|}$ where |·| denotes the set cardinality and *R* and *S* is the reference and predicted masks respectively. DSC is 0 for no overlap and 1 for perfect overlap. It is related to IoU or Jaccard, ${\rm{DSC}}=\frac{2\cdot {\rm{IoU}}}{1+{\rm{IoU}}}$.

#### Precision (*p*), recall (*r*) and F_*β*_ score

These measures evaluate the fraction of correctly predicted instances. Given a number of true instances #GT (ground-truth bounding boxes or pixels in image segmentation) and number of predicted instances #Pred by a method, precision is the fraction of predicted instances that were correctly found, $p=\frac{\#{\rm{TP}}}{\#{\rm{Pred}}.}$ where #TP denotes number of true positives and recall is the fraction of ground-truth instances that were correctly predicted, $r=\frac{\#{\rm{TP}}}{\#{\rm{GT}}}$. Ideally, the best methods should have jointly high precision and recall. F_*β*_-scores gives a single score to capture this desirability through a weighted (*β*) harmonic means of precision and recall, ${{\rm{F}}}_{\beta }=(1+{\beta }^{2})\cdot \frac{p\cdot r}{({\beta }^{2}\cdot p)+r}$.

#### Segmentation score

Similar to detection, semantic segmentation accuracy was also measured with a combined weighted score, ${{\rm{score}}}_{{\rm{s}}}=0.75\cdot [0.5\cdot ({{\rm{F}}}_{1}+{\rm{J}})]+0.25\cdot {{\rm{F}}}_{2}$. Compared to DSC, Jaccard, F2 scores alone, the proposed score allows for moderate bias to be placed on recall (*r*) with behaviour intermediate between *F*_1_ and *F*_2_, (Suppl. Fig. 6).

### Evaluation criteria for the out-of-sample generalization

We define out-of-sample generalization of artefact detection as the ability of an algorithm to achieve similar performance when applied to a different imaging dataset that may differ in imaging modality and acquisition protocol but contain the same set of imaging artefact classes. To assess this, participants applied their trained methods to data collected from a sixth institution whose images were neither included in the training nor test data of the detection and segmentation tasks. Assuming that participants applied the same trained weights, the out-of-sample generalization ability was estimated as the mean deviation between the mAP score of the detection and out-of-sample generalization test datasets of each class *i* for deviation greater than a tolerance of $\{0.1\ast {{\rm{mAP}}\,}_{{\rm{d}}}^{i}\}$.

1 $${{\rm{dev}}}_{{\rm{g}}}=\frac{1}{N}\sum _{i}{{{\rm{dev}}}_{{\rm{g}}}}^{i}$$

2 $${{{\rm{dev}}}_{{\rm{g}}}}^{i}=(\begin{array}{ll}0, & {\rm{for}}|{{{\rm{mAP}}}_{{\rm{d}}}}^{i}-{{{\rm{mAP}}}_{{\rm{g}}}}^{i}|/{{{\rm{mAP}}}_{{\rm{d}}}}^{i}\le 0.1\\ |{{{\rm{mAP}}}_{{\rm{d}}}}^{i}-{{{\rm{mAP}}}_{{\rm{g}}}}^{i}|, & {\rm{for}}|{{{\rm{mAP}}}_{{\rm{d}}}}^{i}-{{{\rm{mAP}}}_{{\rm{g}}}}^{i}|/{{{\rm{mAP}}}_{{\rm{d}}}}^{i} > 0.1\end{array}$$

The deviation score dev_g_ can be either positive or negative, however, the absolute difference should be very small (ideally, dev_g_ = 0). The best algorithm should have high mAP_g_ and high mAP_d_ but a very low dev_g_ (→0). This is because the methods should be robust to perform equally well in both seen and unseen datasets. In Eq. (1), ${{{\rm{dev}}}_{{\rm{g}}}}^{i}=0$ is empirically assigned to an acceptable level of mAP fluctuations (≤0.1) while larger deviations are penalized with the estimated mAP difference score.

The participants were ranked using the weighted ranking score for out-of-sample generalization as ${{\rm{score}}}_{{\rm{g}}}=1/3\cdot {\rm{Rank}}({{\rm{dev}}}_{{\rm{g}}})+2/3\cdot {\rm{Rank}}({{\rm{mAP}}}_{{\rm{g}}})$ where Rank(mAP_g_) denotes the rank of a participant when sorted by mAP_g_ in ascending order.

### Evaluation criteria for clinical relevance

Not assessed as part of the challenge, we additionally evaluated the clinical translation relevance assessments to identify the clinical applicability of the submitted methods. The factors that are critical for clinical relevance are based on (i) accuracy, (ii) consistency of prediction, and (iii) computational efficiency. The consistency of performance is evaluated by considering the standard deviation of various metrics normalized by the mean score. We consider a method to be significant if the standard deviation is low across all artefact classes. Detailed evaluation criteria of this analysis are described in Supplementary Note II.

## Results

### Detection performance

In total 23/29 teams submitted to the detection challenge task of which 15 teams also submitted out-of-sample generalization results and had valid mAP-IoU ratio checks for detection (see Section Performance Evaluation Criteria above). Table 1 summarizes the main aspects of each approach, including the deep learning architecture, processing time, required computation resource, and motivation of design choices. All methods are built upon deep learning advances as discussed in Supplementary Note III. The vast majority of methods aim to improve accuracy by combining the predictions from multiple trained networks based on popular state-of-the-art baseline architectures such as Mask R-CNN^15^ or cascade R-CNN^17^. We refer to these methods as “Ensemble”, i.e., final results are combined from multiple networks trained on the same task, or “symbiosis”, i.e., exploiting mutually beneficial features learnt from different tasks. The remaining methods are either focussed on improving the capture of contextual information across spatial scales to better apprehend the spatial statistics of individual artefact types, for example, by maintaining spatial prediction ("ShufanYang") or concentrate on increasing the number of size-aware training and prediction strategies (XiaokangWang","swtnb" as shown in Table 1 and detailed in Supplementary Note IV). We refer to these methods as “Context” aware detection methods. In general, the use of fewer networks with single-stage prediction (e.g. RetinaNet^16^) resulted in the shortest inference time.

**Figure 2:**
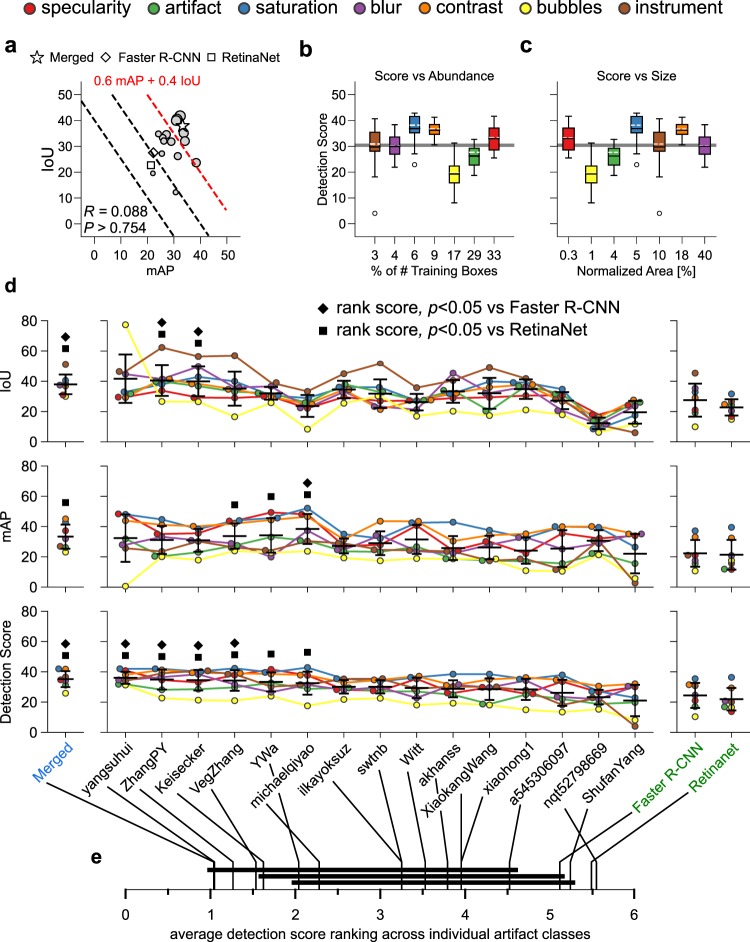
Detection performance of EAD participants on the test dataset. (**a**) Plot of mean IoU vs mAP, (see also Table 2). Each point represents a team plotted with decreasing marker size with decreasing order of detection score, score_d_. Points that lie along the same black dashed lines have the same score_d_ but show a different trade-off between mAP and IoU. The red line highlight the best performing teams (to the right). Box-plot of test detection score, score_d_ for individual artefact classes sorted by increasing % of training boxes, (**b**) and by the normalized box area (area after box width and height have been normalized by the respective image width and height) for all images in the training dataset, (**c**) White and black horizontal lines indicate the mean and median of boxes. Whiskers are plotted at 1.5 × inter-quartile range of upper and lower quartiles. (**d**) Error bars and swarm plots of IoU (top), mAP (middle) and the final detection score, (score_d_, bottom) for each team and baseline methods. Teams are arranged by decreasing score_d_. Error bars show ± 1 standard deviation relative to the mean score across artefact classes. For better visualization, points are adjusted such that they do not overlap in the *x*-axis. Filled square and diamond markers mark teams whose average ranked performance is significantly different to respective Faster R-CNN and Retinanet methods following Friedman Bonferroni-Dunn post-hoc testing with *p* < 0.05. (**e**) Average rank performance of individual methods considering artefact classes independently with detection scores. Solid black lines join methods with no significant rank difference with Friedman Nemenyi post-hoc analysis and *p* < 0.05. Color bars (**b,c**) and color points (**d**) constituting of red, green, blue, violet, orange, yellow and brown represent specularity, artefact, saturation, blur, contrast, bubbles, and instrument classes, respectively.

We trained an in-house Faster R-CNN^20^ and RetinaNet^16^ baseline networks pre-initialized with ImageNet weights **(**Supplementary Note II**)** in order to determine the significance of the submitted results compared to the existing neural networks. Algorithms with performance better than these baseline methods can be recognized as contributors towards technical advancement above current established state-of-the-art computer vision detection methods. In addition, we created a super detector by keeping the detected bounding boxes that overlap with the majority of submissions to test if the combined predictions from all these methods (ensembled technique) yield improvements and to assess complementarity between participant methods (Suppl. Figs. 7, 8, and Suppl. Note II). As expected almost all teams reported mean global scores higher than both of our baseline detection methods. Top-ranked “yangsuhui” also scored higher than the “Merged” super detector (Fig. 2a, Table 2). However, the mean detection score (score_d_) does not reflect large variation in the performance across individual artefact classes. For all submissions, saturation, contrast and specularity classes exhibit consistently better performance and artefact and bubbles consistently worse performance relative to the reported global mean score_d_, (Fig. 2b–d). Interestingly, the worst performing classes (artefact and bubbles) did not necessarily have the fewest number of annotated boxes but both were small in area with large overlaps with boxes of other classes (Suppl. Fig. 4). The Jonckheere-Terpstra test^21,22^ (Supplementary Note II) statistically validates this observation. We found insufficient evidence of dependence between score_d_ and artefact class when sorted by increasing number of training annotation boxes (even when artefact and bubbles classes were excluded, *p*-value > 0.0612, Fig. 2b) but evidence of positive dependence between score_d_ and artefact class when sorted by mean artefact size (*p*-value < 0.0079, Fig. 2c).

**Figure 3:**
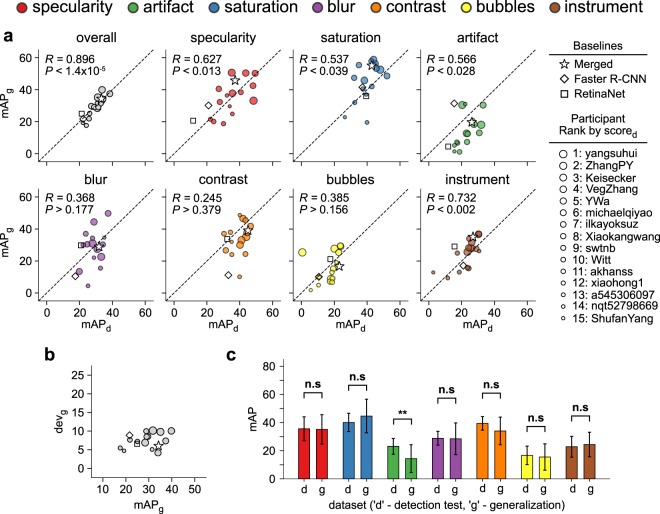
Out-of-sample generalization of participant detection methods. (**a**) Plot of detection (mAP_d_) and generalization (mAP_g_) mAP values per team: Overall (gray) and per artefact class (colored, legend is provided on the top). The black dashed line represents the ideal identity line. *R* denotes Pearson’s *R* correlation. *P* denotes the *p*-value of the null hypothesis being that the slope of the least-squares linear line is zero. (**b**) Plot of deviation score (score_d_) and generalization mAP, (mAP_g_) per team. Team markers in panel (a,b) are plotted large to small with decreasing detection score. score_d_. (**c**) Paired bar plots of mean team detection and generalization mAP scores are denoted by ‘d’ and ‘g’, respectively, for each artefact class. Error bars show ± 1 standard deviation of team scores relative to the overall team mean score shown by each bar. Paired *t*-test was used to test for difference in mean, n.s. - no significance, *p < 0.05, **p < 0.01. In all panels, the same color scheme is used to color individual artefact classes. Color points in (**a**) and color bars in (**c**) constituting of red, green, blue, violet, orange, yellow and brown colors represent for specularity, artefact, saturation, blur, contrast, bubbles, and instrument classes, respectively. Similarly, gray colored points in (**a,b**) are used to represent overall performance of each team. Also, star, diamond and square are used to represent baseline methods in comparison.

Strikingly, a more detailed analysis of the class-specific performance, captured by mAP and IoU scores (Fig. 2d), reveals that higher score_d_ did not imply jointly higher mAP and IoU scores (Fig. 2a). There was a strong evidence of the trade-off between mAP and IoU (c.f. top 6 participants, Fig. 2d, Table 3) because of the implemented mAP-IoU ratio check. Furthermore, all teams suffer from significant class-specific performance variability with large error bars (Fig. 2d). Applying non-parametric Friedman-Nemenyi analysis of variance and post-hoc analysis to assess statistically significant difference in ranked performances^6,23^. The top-ranked by score_d_ teams, “yangsuhui”, “ZhangPY” and “VegZhang” demonstrate consistent improvement (score_d_) over bottom ranked “nqt52798669”, “ShufanYang” and both baselines across artefact classes, (Fig. 2d,e, Supplementary Note II). However these teams were not equally good with respect to mAP and IoU metrics where notable shuffling of team ranks could be observed, for example with “michaelqiyao”, (Suppl. Fig. 9). By taking only consensus detections, the “Merged” detector maintains low variation across artefact classes and demonstrates jointly high rank performance across all metrics. Notably boxes used by the “Merged” detector does not all come from top-ranked teams as might be expected but rather a mixture of 7 high and low-ranked teams; 1st yangsuhui, 5th YWa, 8th swtnb, 9th Witt, 11th XiaokangWang, 12th xiaohong1 and 14th nqt52798669. The same teams that contributed the most prevalence in the detection ‘test’ dataset also contributed the most in the ‘out-of-sample generalization’ dataset. Teams had low contribution if an alternative method produced the same consensus box predictions but with higher confidence. Our results therefore strongly suggest unexploited complementarity between individual training approaches. Interestingly, this can be visually observed in Suppl. Fig. 10 and is suggestive of potential unique differences in the training strategies used by teams that differentially exploit different clinical aspects of endoscopic artefacts; Cascade R-CNN with targeted class balancing of 1st yangsuhui, targeted artefact size by combining prediction from Mask R-CNN and YOLOv3 of 8th swtnb, size specific bounding box augmentation approach of 11th XiaokangWang and image patch-based deep learning approach of 12th xiaohong1.

Visual inspection of detection produced by selected top- and bottom- ranking teams additionally suggest most methods successfully localise artefact containing regions but higher ranking methods tend to better classify and to resolve spatial overlap between bounding boxes. Thus, we conducted a class confusion matrix analysis, (Suppl. Fig. 11, Supplementary Note II). Our results suggest the occurrence of spatial overlap between bounding boxes (Suppl. Fig. 4) decreases the performance of the classifier. When two or more artefact classes are “confused” and compared, neural network predictions tended to favour the class with more training annotations and occupies a larger spatial area. This respectively reflects the data-driven nature of neural network architectures and the preference of non-maximum suppression postprocessing to retain larger regions. For example, across all methods, artefact is confused with specularity, bubbles is confused with both artefact and specularity but as specularity has more annotations it has the best performance. Similarly, there is confusion of instrument with saturation, artefact and specularity due to the metallic or plastic surface of endscopic instruments. Here the larger size of instrument relative to imaging artefact facilitates better detection performance with higher IoU versus specularity but the relatively smaller number of instrument training occurrences produces worse mAP (Fig. 2b–d). These observations appear shared across compared baseline, 6 top-ranking and 3 bottom-ranking methods which all show a similar global confusion matrix pattern (Suppl. Fig. 11a). Consistently 25–30% of all boxes are misclassified (Suppl. Fig. 11b). Surprisingly the best classifiers were not all top-ranked score_d_ methods. Instead the best classifying methods “ZhangPY” (2nd, Mask R-CNN), “Keisecker (3rd, ensemble Retinanet), “ilkayoksuz” (7th, Retinanet, 5-fold validation) and “XiaokangWang” (11th, size-specific augmentation) utilised effective strategies to more accurately estimate box size and location (Fig. 2d, Suppl. Fig. 9) and generate the most number of correct predictions for the total number of predicted boxes, (Suppl. Fig. 12). It is to be noted that these methods were not all two-stage detectors.

Finally, we analyzed per image performance using F1-score in Suppl. Fig. 13a. Rather than there being certain image subsets that were easier for particular teams as might be hypothesized from the complementarity in bounding box predictions, we observed that all teams in general found the same images to be hard (almost no artefacts were detected) or easy (almost all artefacts detected), (Suppl. Fig. 13b). The top 24 ‘easy’ and ‘hard’ images (Suppl. Fig. 13c) provide a concise visual overview of the limitations of current neural network methods which are biased towards large well-defined objects and cannot handle spatial overlap involving small objects irrespective of the amount of available training annotation. Typically the ‘easy’ images belong to single class and possess artefacts with well-defined image boundaries and minimal overlap. Conversely the ‘hard’ images consisted of multi-class artefacts and spatially overlapped boxes with ambiguous artefact classes. This limitation for current detection is likely due to the use of IoU for determining a positive match between predicted and reference boxes both in training and non-maximum suppression (NMS) post-processing. Despite the higher frequency of spatial overlap involving smaller artefacts, the spatial overlap between larger artefacts have much higher IoU, (Suppl. Fig. 4c). At the same time, small objects are more susceptible to small errors, but due to their size this can eventually lead to a null IoU. Consequently, small objects are more likely to produce reduced training signals and be suppressed during NMS.

### Out-of-sample detection generalization performance

The deviation between reported mAP on the detection test and the out-of-generalization dataset obtained from a separate sixth institute was used to analyse the out-of-sample generalization performance. Submissions for the same 15 teams we analysed for detection performance (Table 2, Fig. 3). It is encouraging to note that we observed a strong, significant linear correlation (Pearson’s *R* = 0.896) between global detection (mAP_d_) and generalization (mAP_g_) mAP for all teams independent of rank. The correlation between the mAP_d_ and mAP_g_ scores also was also observed across teams for each artefact class though not always linearly. Blur, contrast and bubbles were the classes that tested non-significant for linear correlation, (Fig. 3a). Further evidence was provided by a flat deviation score, (dev_g_,Supplementary Note II) with respect to mAP_g_ across all teams, (Fig. 3b). Finally paired *t*-test showed non-significant difference in mean mAP between detection and generalization in all classes except for artefact, (Fig. 3c).

**Figure 4:**
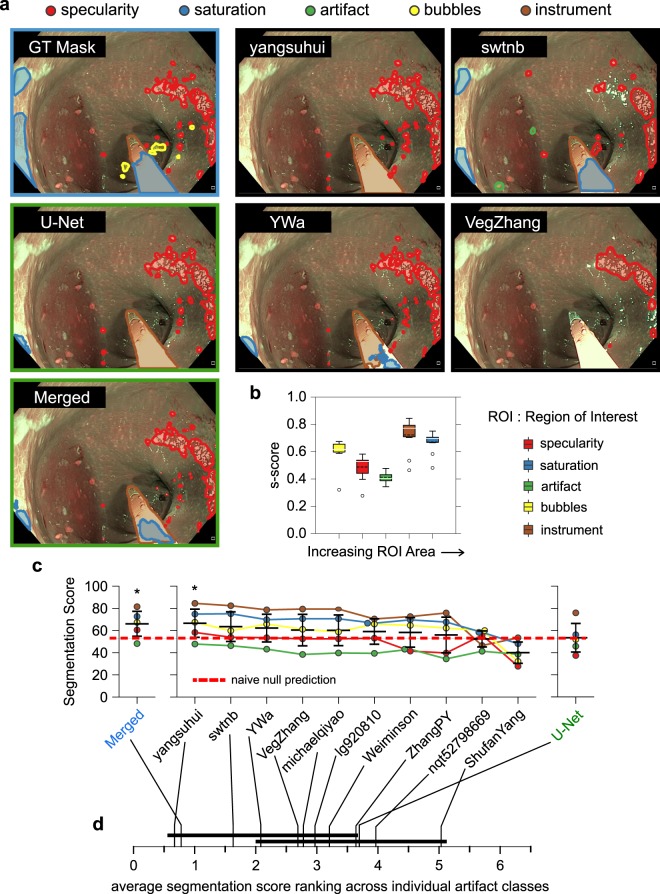
Artefact semantic segmentation performance. (**a**) Segmentation masks predicted by top ranked methods (black border) for a representative image from the test set. Ground truth (GT) mask (top-left and blue bordered) and baseline methods (middle and bottom left, green bordered) are shown for comparison. (**b**) Box plot of s-score and artefact class over all teams. Classes are plotted in increasing order of region-of-interest area. Whiskers are plotted at 1.5 × inter-quartile range of upper and lower quartiles. Outliers are plotted as black points. **c**. Error and swarm plots of s-score. Teams are ordered by decreasing mean s-scores. Error bars show ± one standard deviation of class-specific scores relative to the global mean score for each team. Red dashed line plots the s-score if blank segmentation masks were predicted. ‘*’ denotes statistical difference (*p* < 0.05) in ranked performance relative to the U-Net baseline following Friedman with Bonferroni-Dunn posthoc testing. (**d**) Average s-score rank performance of individual methods considering artefact classes independently. Solid black lines join methods with no significant rank difference following Friedman Nemenyi post-hoc analysis with *p* < 0.05. Colored annotation regions in (**a**), color bars in (**b**) and color points in (**c**) constituting of red, green, blue, violet, orange, yellow and brown represent specularity, artefact, saturation, blur, contrast, bubbles, and instrument classes, respectively.

### Semantic segmentation performance

Table 4 summarizes participated team methods and scores with teams sorted in descending order of final semantic score (s-score; see Supplementary Note II). Similar to the detection task, all submitted methods build upon deep learning advances discussed in Supplementary Note III. However, we observed a large variation in performances. Here, we established a baseline by training an in-house U-Net network^24^. In addition, we also created ‘super’ segmentations by retaining the consensus segmentations across all participants (for details please refer to Suppl. Note II, Suppl. Fig. 14) to test if an ensemble of predictions yield improvements. This approach also reveals the extent of complementarity between participants. Overall, the ensemble method of “yangsuhui” (0.6654) and detection enhanced segmentation of “swtnb” (0.6348) achieved the best s-scores. Notably, both methods try to better capture the underlying data variation by combining feature maps obtained from different backbones. Spatial pyramid pooling of features by “michaelqiyao” in PSPNet^25^ to better capture the varying sizes of individual region-of-interest (ROIs) of artefact classes (Suppl. Fig. 3c,d) in the EAD2019 dataset was also an effective strategy for single stage neural networks. Alternative strategies such as cascaded mask R-CNN and mask aided R-CNN appear to fare worse. Despite the overwhelming popularity of U-Net^24^ for semantic segmentation in biomedical imaging, our baseline U-Net and the UNet-D of “ShufanYang” using ResNet50 backbone were the worst performing, ~15–30% lower than the top method of “yangsuhui”.

Visual observation of predicted segmentation masks indicate that the four top ranked methods are capable of capturing most salient imaging artefacts. However, similar to the detection task, it can be observed in Fig. 4a,c that there is significant variability in performances across individual classes and difficulty to handle class overlaps. Overall the smaller the mean connected area of an artefact, the worse the segmentation peromrance measured by the s-score. Saturation and instrument classes were the best performing whilst artefact and specularity were the worst performing classes, (Fig. 4b). While the segmentations of bubbles did cause difficulties, the accuracy of segmenting bubbles compares with the segmentation accuracy of the classes saturation and instrument. The presence of connected regions of specularity and artefacts that enforce them to form more irregular shapes is one plausible explanation. Irregularly shaped regions cannot be handled by standard convolution kernels that impose a regular sampling grid^26^. Alternative metrics, Jaccard, DSC, overlap and F2 all show similar results, (Suppl. Fig. 16). Remarkably, the observed class-specific performance variation is shared by all individual teams with the exception of “nqt52798669”, (Fig. 4c). The method submitted by “nqt52798669” is the notable exception. The strategy of maximally exploitingg feature map combination from different neural network layers corresponding to different object size through deep layer aggregation^27^ is unique in this approach. While this explains the very small error bar, it fails to explain its low performance (2nd last). The method of yangsuhui consistently ranked first across the majority of metrics, s-score, Dice coefficient and Jaccard (Fig. 4c, Suppl. Fig. 15a,b). However it statistically only performed better than the bottom two methods (see 4d). Quantitatively, “yangsuhui” was even better than the consensus produced “Merged” segmentation (2nd best) whose segmentations were shared equally by “yangsuhui” and “swtnb”(Fig. 4d, Suppl. Fig. 14c, Suppl. Fig. 15a,b).

Aside from good overlap, it is also important to maximize the number of positive predictions and minimize the number of false positives. We observe a positive correlation between the proportion of positive pixel-wise segmentation (PPV or precision, see Supplementary Note II) and the s-score (Pearson’s *R* = 0.84, *p*-value < 0.0025). While the ranking with respect to these different measures differs, it is still correlated (Spearman’s *R* = 0.67, *p*-value < 0.033). Third ranked “YWa” by s-score is now first ranked with PPV = 0.904, >2% higher than “yangsuhui” (first ranked by s-score), (Suppl. Fig. 15e, Table 4). In addition, despite lower in s-score, the artefact class is better than specularity under PPV (Suppl. Fig. 16e). Recall shows similar results, even though positive correlation with s-score is less strong (Pearson’s *R* = 0.64, *p*-value < 0.0456) and correlated, different ranking (Spearman’s *R* = 0.70, *p*-value < 0.025). Second ranked “swtnb” is now best ranked which may explain its prevalence in the “Merged” segmentation. Notably the variation in the recall of individual classes (except specularity) across all methods is low and stable compared to other measures, (Suppl. Fig. 16). This suggests participant methods primarily improved only the per pixel accuracy of predicted regions but that the predicted regions are likely only the same regions for all methods. The actual number of distinct ground-truth regions that could be recalled did not necessarily increase across methods. For example, no method could predict the presence of bubbles in Fig. 4a and all aggregated team predictions could not predict the full saturated region, (Suppl. Fig. 10a). This is supported by the fact that all teams, in general, found the same images ‘easy’ or ‘hard’ to segment (Suppl. Fig. 17). As with detection ‘easy’ images were typically single class, larger connected regions of regular geometry and minimal overlap between classes. Meanwhile ‘hard’ images have highly spatially overlapping classes, cover regions of discontinuous image intensity and exhibit regions of different small and large sizes. Taken together, we found the top-ranking neural network methods were able to segment artefacts, however, the performances are highly variable across classes. They are most effective for artefacts of large area and regular convex polygonal geometries. It must be cautioned that current evaluation metrics are imperfect. Due to the natural pixel imbalance with more background (0 values) than foreground pixels (values with 1) in binary class masks, null prediction will result in an s-score of 0.5289 which is comparable to our U-Net baseline when plotted as a red dashed line in Fig. 4c. By extension this inherent pixel imbalance problem is subsequently often overlooked in training neural networks for segmentation.

### Clinical applicability of methods

The critical factors for clinical application are (i) accuracy, (ii) consistency of performance, and (iii) computational efficiency. Whilst accuracy is important and has been the primary consideration in academic publications, we argue that performance consistency is equally important in clinical applications. For example, given the diagnostic implications of two algorithms, one that produces consistent predictions with an acceptable mean accuracy across multiple clinical setups is preferred over the one that has higher accuracy but only for a few fixed clinical setups. Of lesser importance but of significant practical consideration is computational efficiency. The method requiring least computational memory and power is more economical. Further, fast execution enables real-time applications. To objectively evaluate each of the three criteria we conducted detailed clinical applicability analyses by aggregating rankings from multiple metrics (see Supplementary Note II, Tables 5, 6) used in detection and segmentation performance dissections. The final applicability ranking was produced from a weighted average of accuracy, consistency, and efficiency rankings with weights 0.4, 0.5, and 0.1, respectively. In Table 5, the detection method of “ilkayoksuz” is clinically top-ranked despite the 5^*th*^ position by score_d_ whilst “yangsuhui” method is only 11^*th*^ (similar to our baseline “RetinaNet”). It is to be noted that “yangsuhui” is ranked 1^*st*^ on the challenge leaderboard. Among all algorithms, RetinaNet variants (“ilkayoksuz”, “Keisecker”) score highest for all factors followed by Mask R-CNN variants (“ZhangPY”, “swtnb”).

**Table 5:** Clinical applicability ranking of the participants detection methods with appraisal for their accuracy, consistency and computational efficiency.

Team	Accuracy				Consistency				Computational Efficiency					Clin.Rank
	mAP	IoU	Conf.	Acc.	mAP-	IoU-	Gen.	Cons.	Back-	Mult.	GPU	Test	Eff.	
			Score	Rank	std.	std.		Rank	bone	Net		Time	Rank	
yangsuhui	5	5	15	9	16	14	7	14	5	1	5	8	5	11
ZhangPY	7	1	5	2	9	8	5	7	8	8	8	8	9	4
Keisecker	8	2	2	1	4	7	7	5	7	6	3	4	6	2
VegZhang	3	8	7	4	5	13	4	6	13*	13*	14*	13*	11	5
YWa	2	6	11	5	12	1	1	2	13*	13*	14*	13*	11	3
michaelqiyao	1	16	10	10	6	10	2	4	13*	13*	14*	13*	11	6
ilkayosuz	9	4	4	3	1	2	6	1	13*	1	3	6	7	1
XiaokangWang	12	11	1	7	8	12	10	11	4	1	5	7	4	8
swtnb	10	7	6	6	7	9	7	8	13*	13*	14	13*	11	7
Witt	4	12	9	8	10	5	12	9	13*	13*	14	13*	11	9
akhanss	11	9	8	11	11	6	14	12	5	13*	9	5	8	13
xianohong1	16	3	12	13	13	3	15	13	13*	13*	14*	13*	11	14
a545306097	15	10	13	14	15	4	17	15	13*	13*	14*	13*	11	16
nqt52798669	6	17	17	16	2	11	3	3	6	1	10	13*	10	10
ShufanYang	17	14	16	17	17	15	12	16	1	1	6	1	2	17
Faster R-CNN	14	15	3	12	14	16	16	17	1	1	1	3	3	15
RetinaNet	12	13	14	15	3	17	10	10	1	1	1	2	1	11

The final clinical relevance rank is also presented. Baseline Faster R-CNN and RetinaNet methods are also included. The lower the rank the better is the performance.

*Conf. Score: confusion matrix score, Acc. Rank: accuracy ranking, mAP-std.: standard deviation/mean mAP ratio, Gen.: out-of-sample generalization, Cons. Rank: consistency ranking, Mult. Net.: use of multiple trained networks, Eff. Rank: computational efficiency ranking, Clin. Rank: overall clinical relevance ranking *imputed ranks.

**Table 6:** Clinical applicability ranking of the participants segmentation methods with consideration of their accuracy, consistency and computational efficiency.

Team	Accuracy				Consistency				Computational Efficiency				
	s-score	PPV	Recall	Acc. Rank	s-score std.	PPV std.	Recall std.	Cons. Rank	Backbone	Mult. Net	GPU	Eff. Rank	Clin. Rank
yangsuhui	1	2	2	1	2	4	3	2	5	9.5	3	9	1
swtnb	2	7	1	2	5	10	1	6	4	1.0	6	3	3
YWa	3	1	9	5	4	2	5	3	4.25	1.0	14	6	3
VegZhang	4	4	4	3	8	6	10	9	4.25	1.0	14	6	8
michaelqiyao	4	5	3	3	7	5	9	7	1.00	1.0	5	1	5
Ig920810	6	6	8	7	3	3	2	1	4.25	1.0	14	6	2
Weiminson	7	3	6	6	6	1	7	5	4.25	1.0	8	4	6
ZhangPy	9	8	7	9	10	9	8	10	3.00	1.0	14	5	10
nqt52798669	8	9	5	8	1	7	4	4	5.00	9.5	9	10	7
ShufanYang	10	10	11	10	9	8	6	8	2.00	1.0	3	2	9
U-Net	11	11	10	11	11	8	6	8	1	1	1	2	10

UNet is also included in the comparison as the baseline method. The lower the rank the better is the performance.

*PPV: positive predictive value, Acc. Rank: accuracy ranking, s-score std.: standard deviation/mean s-score ratio, PPV. std.: standard deviation/mean PPV ratio, Recall std: standard deviation/mean recall ratio, Cons. Rank: consistency ranking, Mult. Net.: use of multiple trained networks, Eff. Rank: computational efficiency ranking, Clin. Rank: overall clinical relevance ranking.

*imputed ranks.

In Table 6, clinical applicability metric revealed that 6^*th*^ ranked “Ig920810” on the segmentation leaderboard has the best performance consistency and ranked 2^*nd*^ in the overall clinical applicability ranking whilst “yangsuhui” is still ranked 1^*st*^. Both “swtnb” and “ZhangPY” used a symbiosis paradigm based on mask R-CNN, however, the combination of mask R-CNN, YOLOv3 and targeted pre-processing used by “swtnb” yielded more accurate results. Also, despite the popularity of U-Net for segmentation in the medical image analysis community, the U-Net-based model of “ShufanYang” was ranked only second last in our clinical applicability analysis. In short, our analysis highlights an ultimate requirement of considering diverse metrics that can capture all critically important aspects for effective clinical translation of these algorithms.

## Discussion

Critical dissections of the submitted methods to the EAD2019 challenge reveal that the application of transfer learning and targeted training strategies such as ensemble technique of “Keisecker” in detection and “yangsuhui” in segmentation yielded in an improved performance compared to the direct application of the state-of-the-art neural networks. However, as suggested in Fig. 2, detection performances critically depend upon the size of an individual artefact class and the extent of spatial overlap irrespective of the amount of training data. *Hypothesis-I*: *the detection performance is inhibited by the use of a single IoU cut-off for determining a positive match between predicted and reference boxes, irrespective of artefact size which also underscores the effect of spatial overlap*.

For out-of-sample generalization, all proposed methods showed potential of delivering similar detection performance on out-of-sample data except for the results on the ‘artefact’ class where the incapability of methods generalizing on large appearance variation observed in artefact is clearly demonstrated despite the presence of large number of training annotations (2^*nd*^ most annotated class, Suppl. Fig. 3). This suggests that large variability in the appearance of artefacts (both intra- and inter-class) present in endoscopy frames is hard-to-generalize which implicates to the fact that training a neural network architecture effectively on endoscopy data for frame artefact detection will require a tremendous amount of samples per artefact class.

For semantic segmentation, results depended upon both artefact sizes and the amount of ground truth labels (foreground masks). Table 4 suggests that improvements in segmentation is mostly due to reduction in number of false positive classifications, i.e., increased precision, whilst only marginal changes in recall suggests that there is a no improvement in false negative classification, i.e., there are regions which are constantly missed by all methods. This can be due to methods failing to capture annotator variance and shape and appearance variability. *Hypothesis-II: the segmentation performance of all methods fail to generalize class imbalance and suffer from an implicit bias to predict ‘0’ due to small areas of artefacts and overabundance of background pixels in binary masks*.

As such, we suggest significant performance gains might be achieved through the implementation of size independent loss functions and metrics that optimally target the spatial overlap between multi-class objects, e.g., use of size-specific multiple IoU thresholds for detection. For segmentation, we suggest the use of deformable convolution strategies^26^ optimized for artefact geometry and incorporating sampling strategies and metrics that can better handle the data imbalance problem such as using uncertainty weighted losses^28^. We also suggest that participants could benefit from exploring pre-sharpening of frames or pre-extraction of attention maps as pre-processing steps to improve performance or build-in attention maps to model scale within the network. Most importantly, our detailed analysis shows that the competition-winning solutions overfit on selected challenge metrics that are often not optimal for practical deployment, particularly in biomedical applications that demand a balance of accuracy, consistency of performance and computational efficiency. We advocate for more holistic ranking procedures which can yield more significant insights for improved technological development that facilitates clinical translation.

As observed in Suppl. Fig. 11, instrument and artefact classes have large collocalization. It might be beneficial in pre- or post-processing to take into account the colocalization of multiple classes to circumvent class imbalance problem, e.g., through finer stratification within artefact classes. However, such context-aware targeted processing of detections were not explored by participants. Nevertheless, the results presented in the EAD2019 challenge surpasses the state-of-the-art methods in both detection and segmentation. Given the complexity of the compiled data in EAD2019 challenge, most methods developed during this challenge has significant strength in clinical usability. It is worth noting that a good trade-off between the mAP and IoU was obtained by the top 15% of the EAD2019 challenge methods. Additionally, an overlap accuracy of over 60% for segmentation method which is nearly 20% more than U-Net architecture widely used in medical imaging field.

Accurate detection, localization and delineation of artefacts can enable efficient end-to-end pipelines for endoscopy quality assessment. This can be used in clinics for training of novice endoscopists and for accessing mucosal surface that has been actually covered during an endoscopic procedure. Additionally, incorporating the detection and segmentation pipeline to reduce the false detection of diseases, such as polyps that are often mistaken with pixel saturation problem in endoscopy videos is another application. It has been shown that identifying and recovering of partially corrupted frames based on detection of artefacts can help restore endoscopic images that can increase the robustness of any post analysis computer vision methods^1^.

## Supplementary information

Supplementary information

## Data Availability

All data generated or analysed during this study are included in this published article and its Supplementary Information files. EAD dataset: 10.17632/c7fjbxcgj9.2. Accompanying software tools is also available for the dataset: https://sharibox.github.io/EAD2019/.
