# Supplementary material for: An objective comparison of detection and segmentation algorithms for artefacts in clinical endoscopy
Source: Sci Rep. 2020 Feb 17;10:2748. doi: 10.1038/s41598-020-59413-5 (PMC7026422; doi:10.1038/s41598-020-59413-5)
Supplement: Supplementary file 1 — Supplementary information [file 41598_2020_59413_MOESM1_ESM.pdf]

An objective comparison of detection and segmentation algorithms for artefacts in clinical endoscopy

Sharib Ali[1,*,+], Felix Zhou[2,+], Barbara Braden[4], Adam Bailey[4], Suhui Yang[6], Guanju Cheng[6], Pengyi Zhang[7], Xiaoqiong Li[7], Maxime Kayser[8], Roger D. Soberanis-Mukul[8], Shadi Albarqouni[8], Xiaokang Wang[9], Chunqing Wang[15], Seiryo Watanabe[10], Ilkay Oksuz[11,20], Qingtian Ning[17], Shufan Yang[16], Mohammad Azam Khan[18], Xiaohong W. Gao[19], Stefano Realdon[5], Maxim Loshchenov[13], Julia A. Schnabel[11], James E. East[4], Georges Wagnieres[12], Victor B. Loschenov[13], Enrico Grisan[14,21], Christian Daul[3], Walter Blondel[3], and Jens Rittscher[1]

[1]Institute of Biomedical Engineering, Department of Engineering Science, University of Oxford, Oxford, UK
[2]Ludwig Institute for Cancer Research, University of Oxford, Oxford, UK
[3]CRAN UMR 7039, University of Lorraine, CNRS, Nancy, France
[4]Translational Gastroenterology Unit, Nuffield Department of Medicine, Experimental Medicine Div., John Radcliffe Hospital, University of Oxford, Oxford, UK
[5]Instituto Onclologico Veneto, IOV-IRCCS, Padova, Italy
[6]Ping An Technology (Shenzhen) Co. Ltd., Shenzhen, China
[7]Beijing Institute of Technology, Beijing, China
[8]Technishe Universität München, Munich, Germany
[9]Department of Biomedical Engineering, University of California, Davis, USA
[10]Department of Bioinformatic Engineering, Osaka University, Suita, Osaka, Japan
[11]School of Biomedical Engineering and Imaging Sciences, King's College London, London, UK
[12]Swiss Federal Institute of Technology in Lausanne (EPFL), Lausanne, Switzerland
[13]A.M. Prokhorov General Physics Institute, Russian Academy of Science, Moscow, Russia
[14]Department of Information Engineering, University of Padova, Padova, Italy
[15]Department of Ultrasound Imaging, Tiantan Hospital, Beijing, China
[16]School of Engineering, University of Glasgow, Glasgow, UK
[17]Department of Automation, Shanghai Jiao Tong University, China
[18]Department of Computer Science and Engineering, Korea University, Seoul, South Korea
[19]Department of Computer Science, Middlesex University, London, UK
[20]Department of Computer Engineering, Istanbul Technical University, Istanbul, Turkey
[21]School of Engineering, London South Bank University, London, UK
[+]these authors contributed equally to this work
[*]sharib.ali@eng.ox.ac.uk

Supplementary Materials and Method

Supplementary Figures

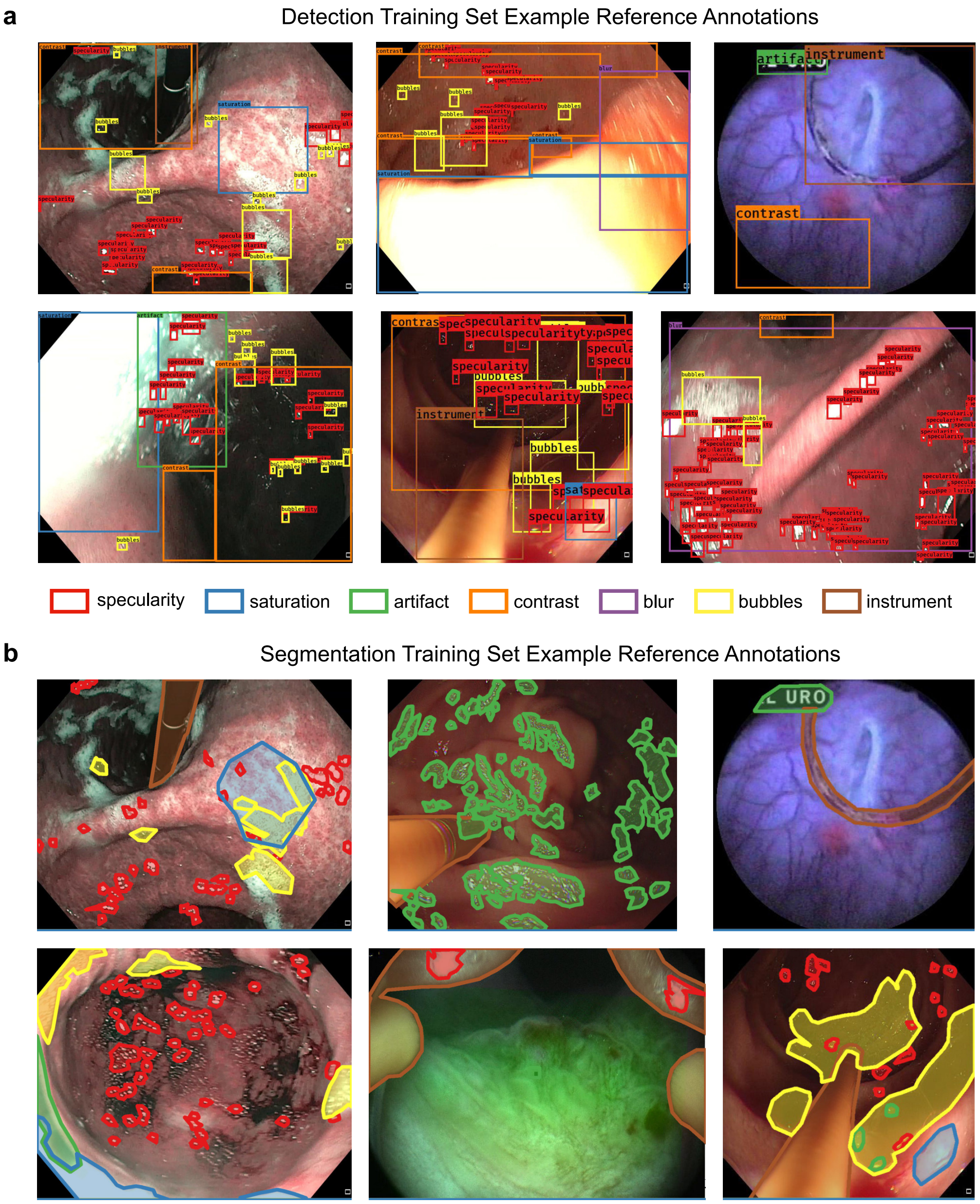

Figure 1. Example reference annotations in the endoscopy artefact detection training dataset. **a**, Bounding box annotations of the 7 classes and **b**, segmentation masks of 5 classes in the EAD challenge training dataset. The segmentation training dataset is a subset of the detection training dataset. All images with segmentation masks have corresponding detection bounding box annotations but not vice versa.

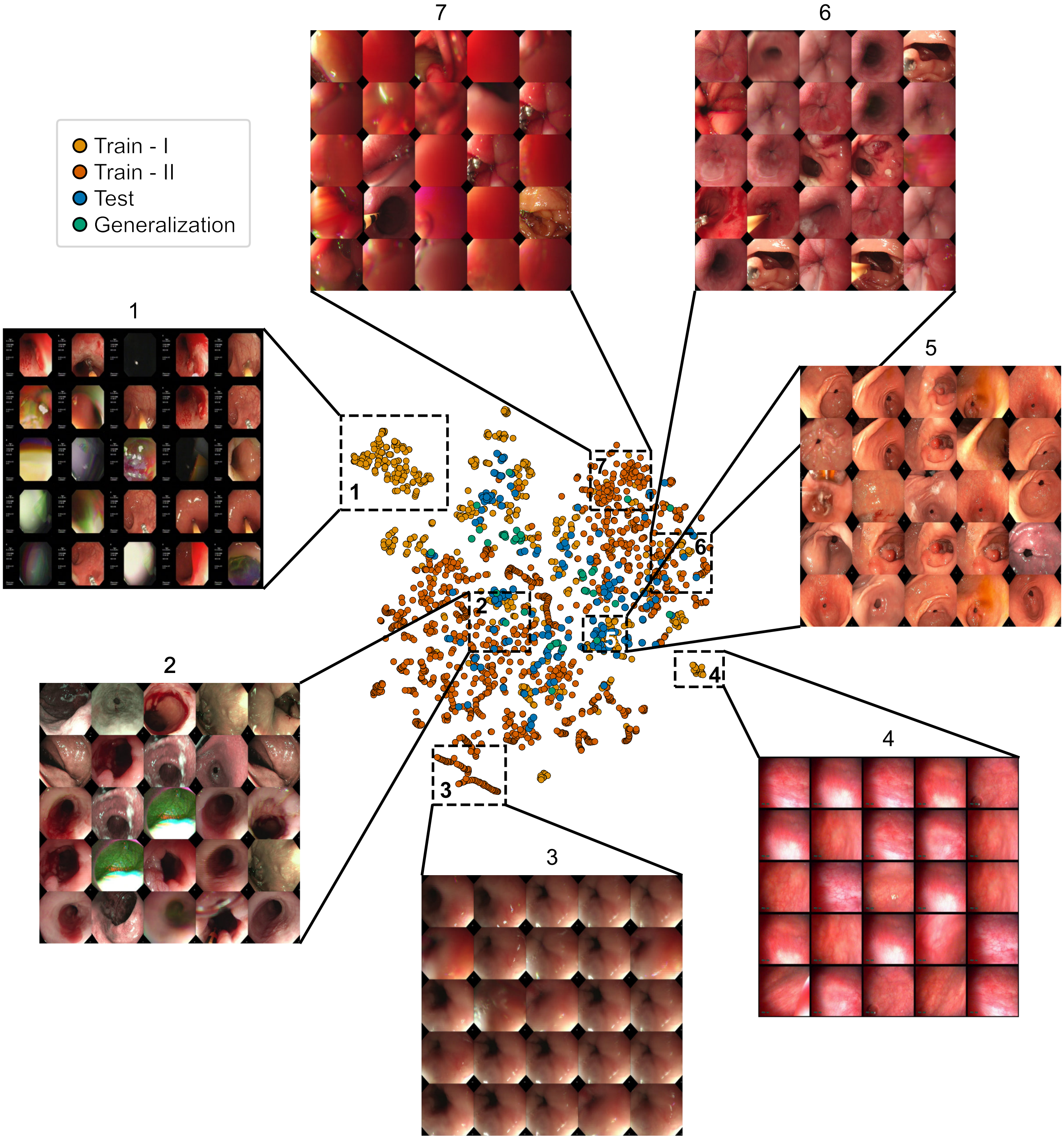

Figure 2. 2D t-SNE embedding of the EAD2019 dataset based on deep autoencoder extracted features. Each point is an image in the EAD2019 dataset. For each of the seven boxed regions (dashed black lines) 25 images were randomly sampled for display in a 5x5 image grid. All images were rescaled to 256 x 256 pixels before applying the autoencoder, see Supplementary Note I for technical details.

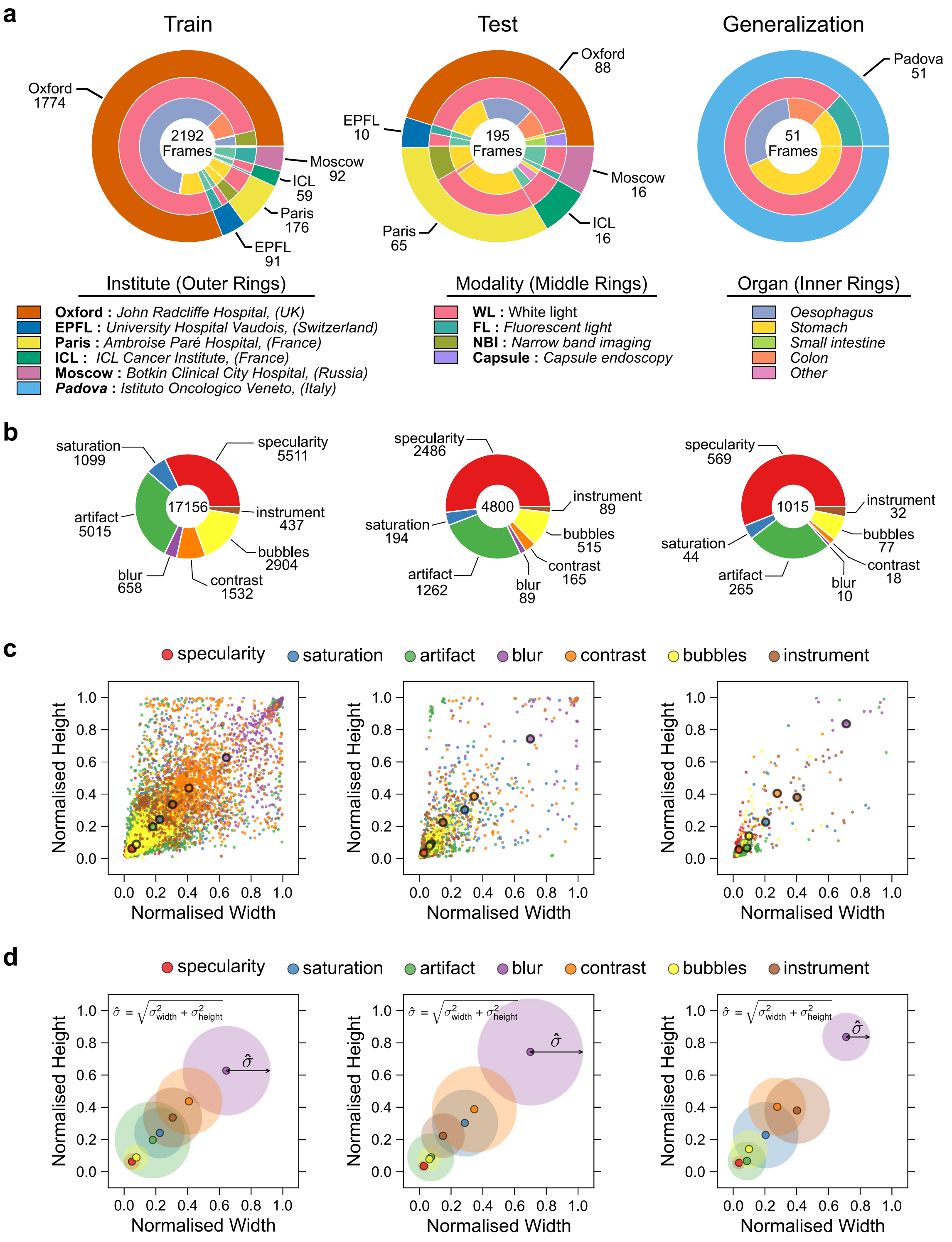

Figure 3. Composition of the endoscopy artefact detection dataset. **a**, Percentage of image frames from each of the 6 data institutions in train, test and generalization datasets by institute (outer ring), imaging modality middle ring and imaged organ (inner ring). ‘Other’ organ indicates frames that cannot be classified into the five main organs including laproscopy and non-organ objects. **b**, Proportion of the total number of artefact bounding boxes (number in pie chart center) for each of the 7 artefact classes in the detection train, test and generalization datasets respectively. **c**, Normalised width vs height relative to source image width and height of annotated ground-truth bounding boxes (per small dot) coloured by class. Larger points plot the mean (width, height) pair of each class. Boxes are primarily square shaped (mean point width:height ≈ 1:1). **d**, Plot of mean box width and height of individual classes of (**c**) and their characteristic standard deviation.

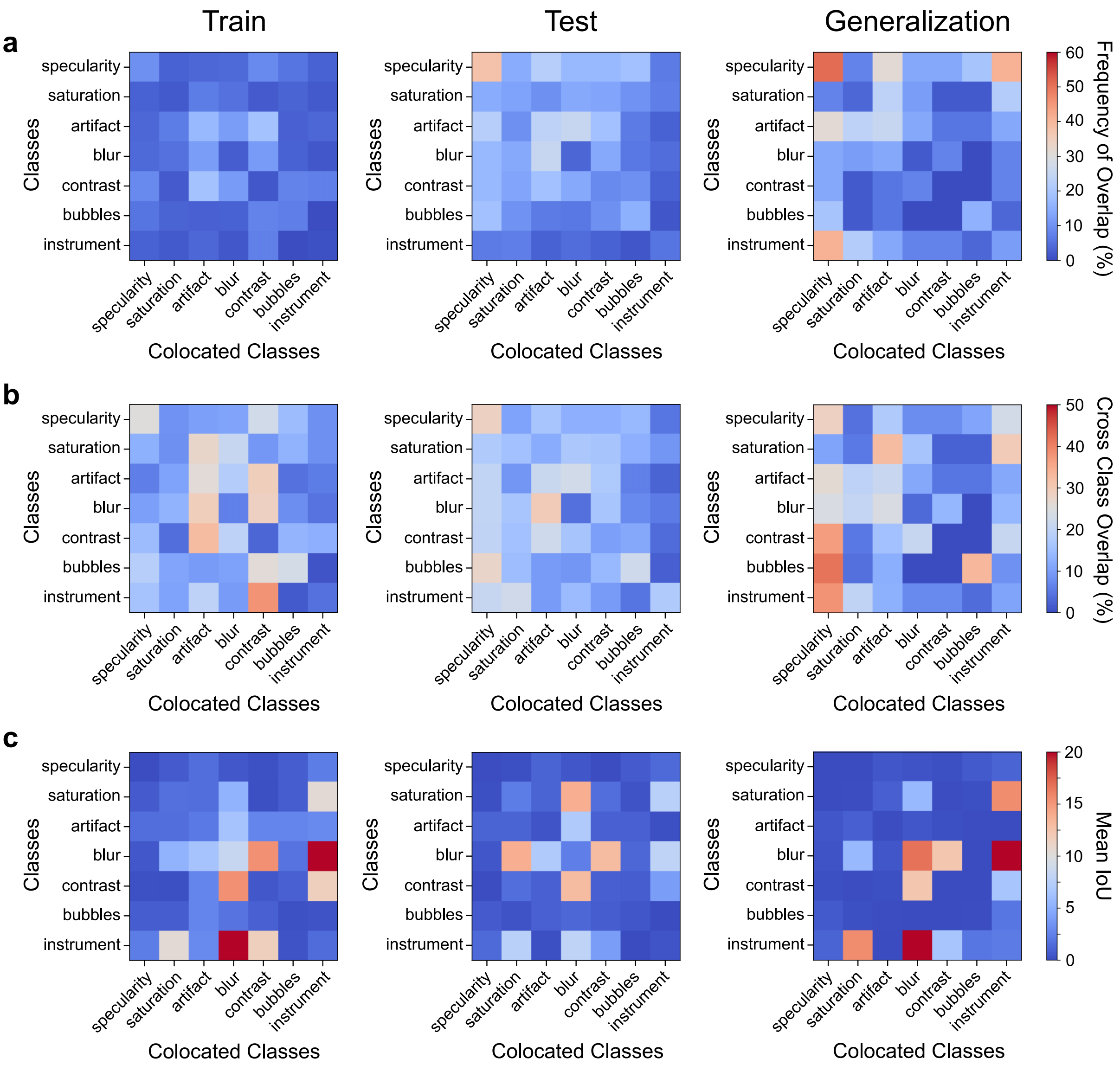

Figure 4. Spatial overlap of ground-truth reference bounding box annotations. **a**, Percentage (%) of boxes in each class (row, y-axis) that spatially overlap (IoU>0) with boxes from each of the 7 classes (column, x-axis). **b**, The distribution of boxes that overlap in each class (row, y-axis) and the proportion of times they overlap with boxes in each of the 7 classes (column, x-axis). It is computed by normalising each row of each matrix in A with their row sum. **c**, The mean IoU of overlapped boxes in each class (row, y-axis) and boxes of each of the 7 classes (column, x-axis). Comparison of (**b**) and (**c**) shows the mean IoU does not reflect the higher frequency of overlap of small artefact classes with other small and large artefacts.

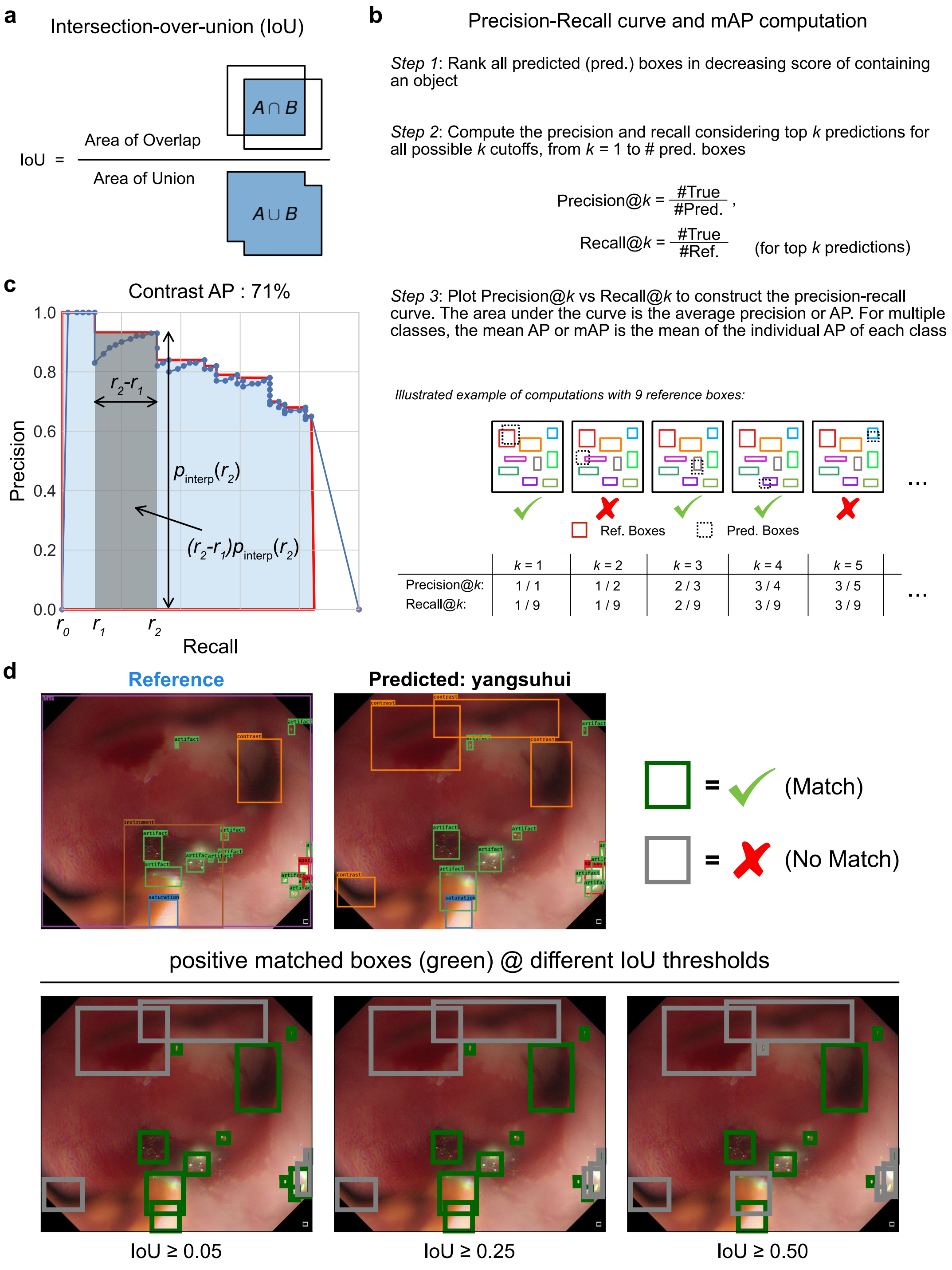

Figure 5. Illustration of the intersection-over-union (IoU) and average precision metrics for evaluating detection performance. **a**, Schematic of IoU. Shaded blue areas illustrate the relevant areas referred to in the equation. **b**, Steps involved in computing the precision and recall for constructing the precision-recall curve and computation of average precision (AP) as the area underneath this curve for assessing ranked detection performance. **c**, Example precision-recall curve for the contrast class for the Faster R-CNN detection baseline. An area element for computing AP is also illustrated. Individual points correspond to each predicted 'contrast' bounding box in the detection test dataset plotted left to right by descending predicted objectness score. **d**, Positively matched (green boxes) and unmatched (grey boxes) predicted boxes by yangsuhui according to different IoU cutoffs for an example test image.

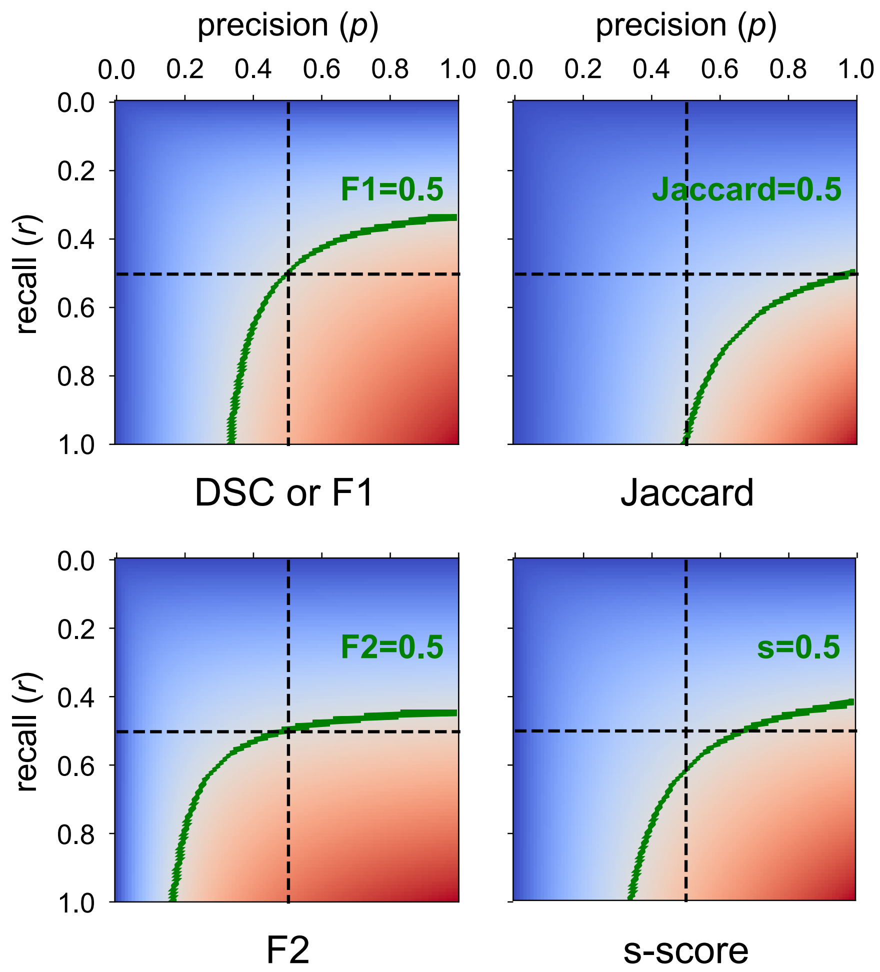

Figure 6. Comparison of the values of different segmentation metrics that take value 0-1 (blue to red) as a two-variable function of precision (p) and recall (r). Black dashed lines mark the $p = 0.5$ and $r = 0.5$ lines. Solid green line show the contour line when the respective metric equals 0.5.

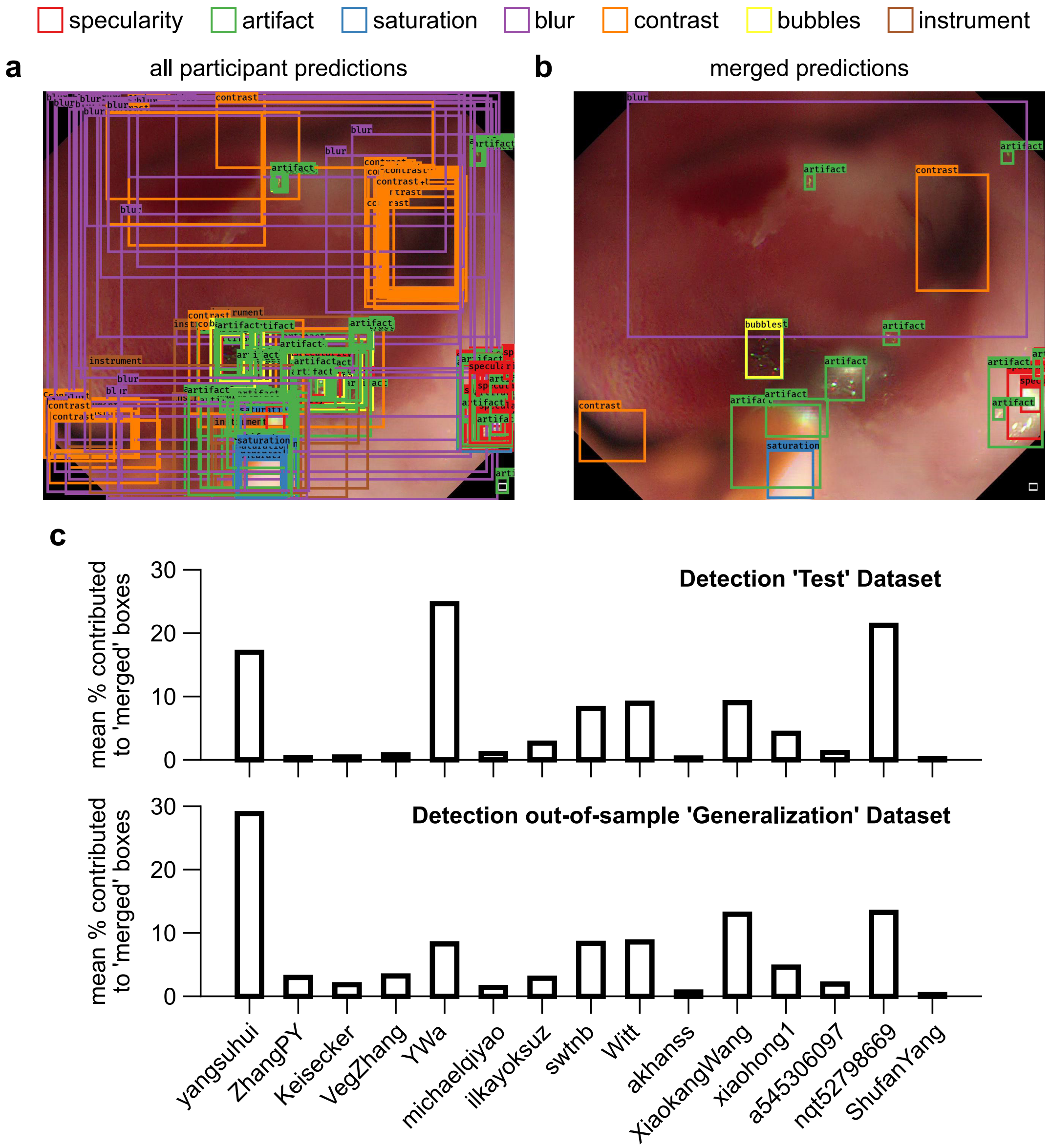

Figure 7. Super detector creation from all team box predictions. **a**, Overlay of all predicted bounding boxes from each of the 15 teams in the challenge. **b**, Final predicted boxes after retaining only the most confident prediction for overlapped bounding box detections. **c**, Mean fraction of bounding boxes contributed by each team to the final merged predicted boxes used by the super detector for the detection test (n = 195 images) and out-of-sample generalization (n = 51 images) datasets.

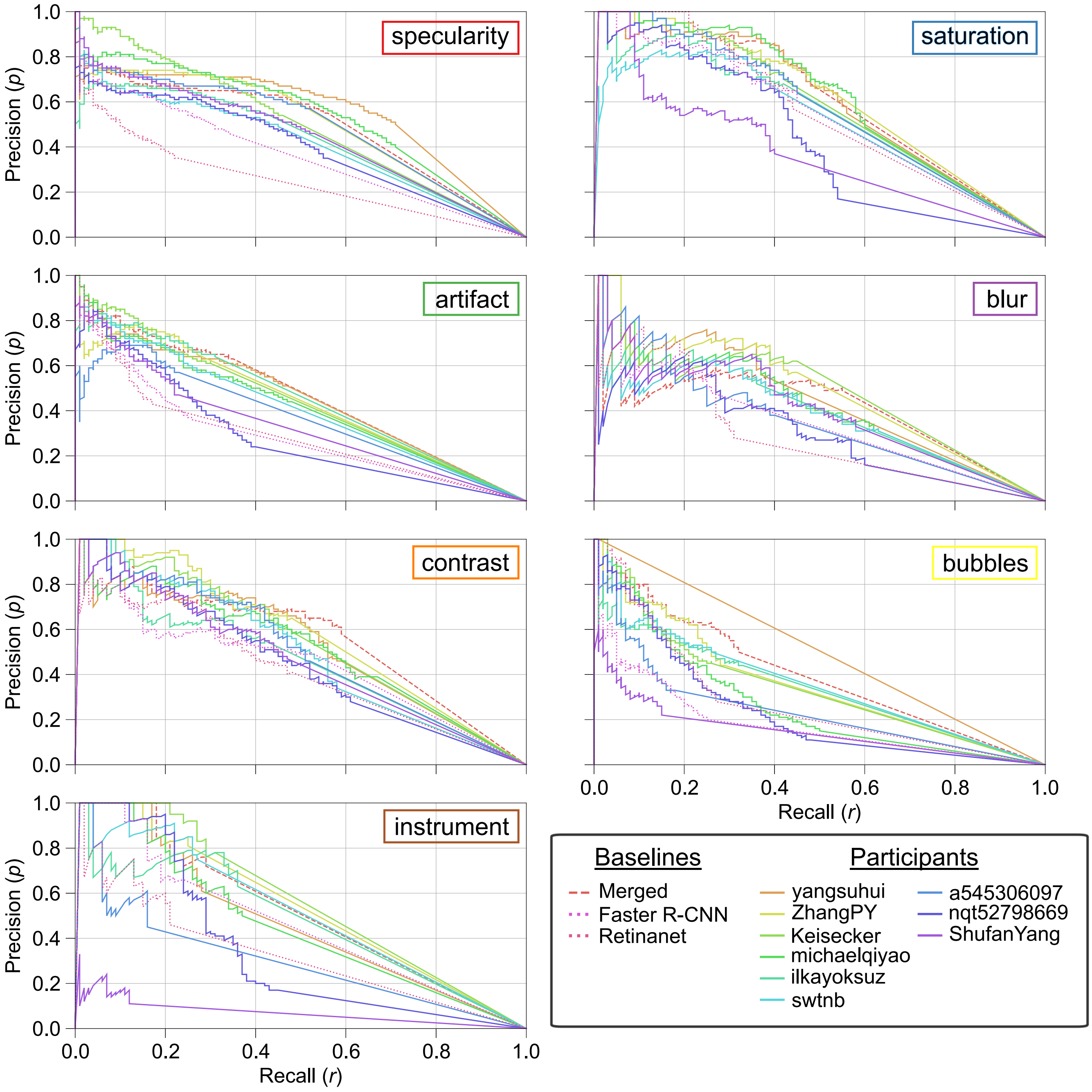

Figure 8. Precision-recall curves of baseline and selected EAD2019 participant artifact detection methods. Long dashed line is the merged detector, dotted lines are the baseline Faster R-CNN and Retinanet methods and solid lines for the top 6 (left) and bottom 3 (right) team detection methods.

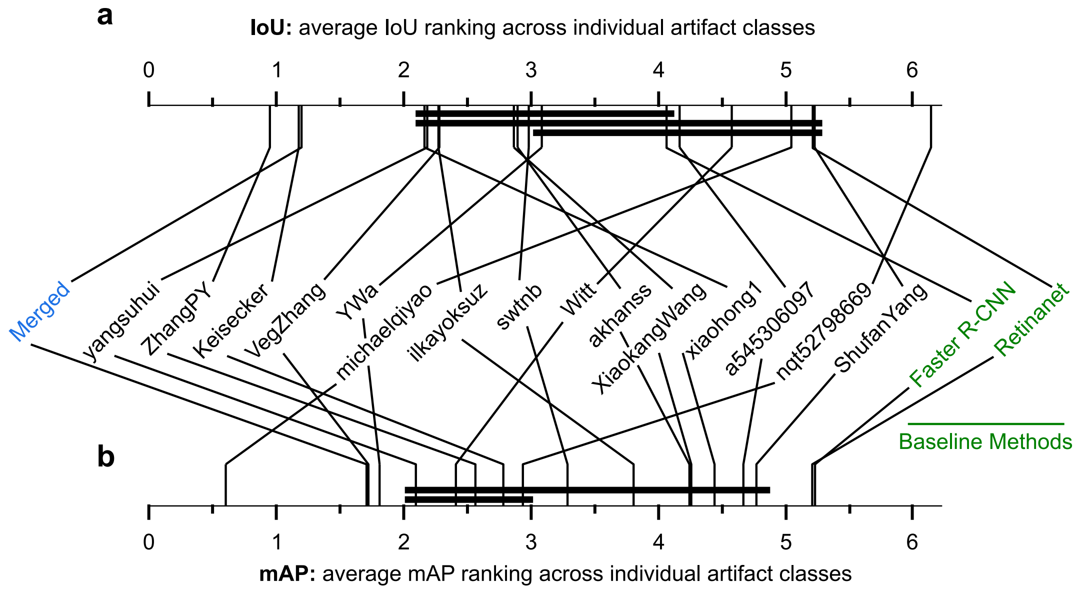

Figure 9. Relative IoU and mAP detection performance of detection methods. Critical difference diagram of IoU, **a** and mAP, **b** detection performance for all individual methods (black font), merged (blue font) prediction baseline and state-of-the-art Faster R-CNN (green font) and Retinanet (green font) baselines. Scale reports the average rank of methods across all artifact classes with respect to each metric. The lower the score the better the performance. Thick black horizontal lines join methods that are not statistically different in rank ($p \leq 0.05$) according to post-hoc Friedman-Nemenyi statistical testing[1].

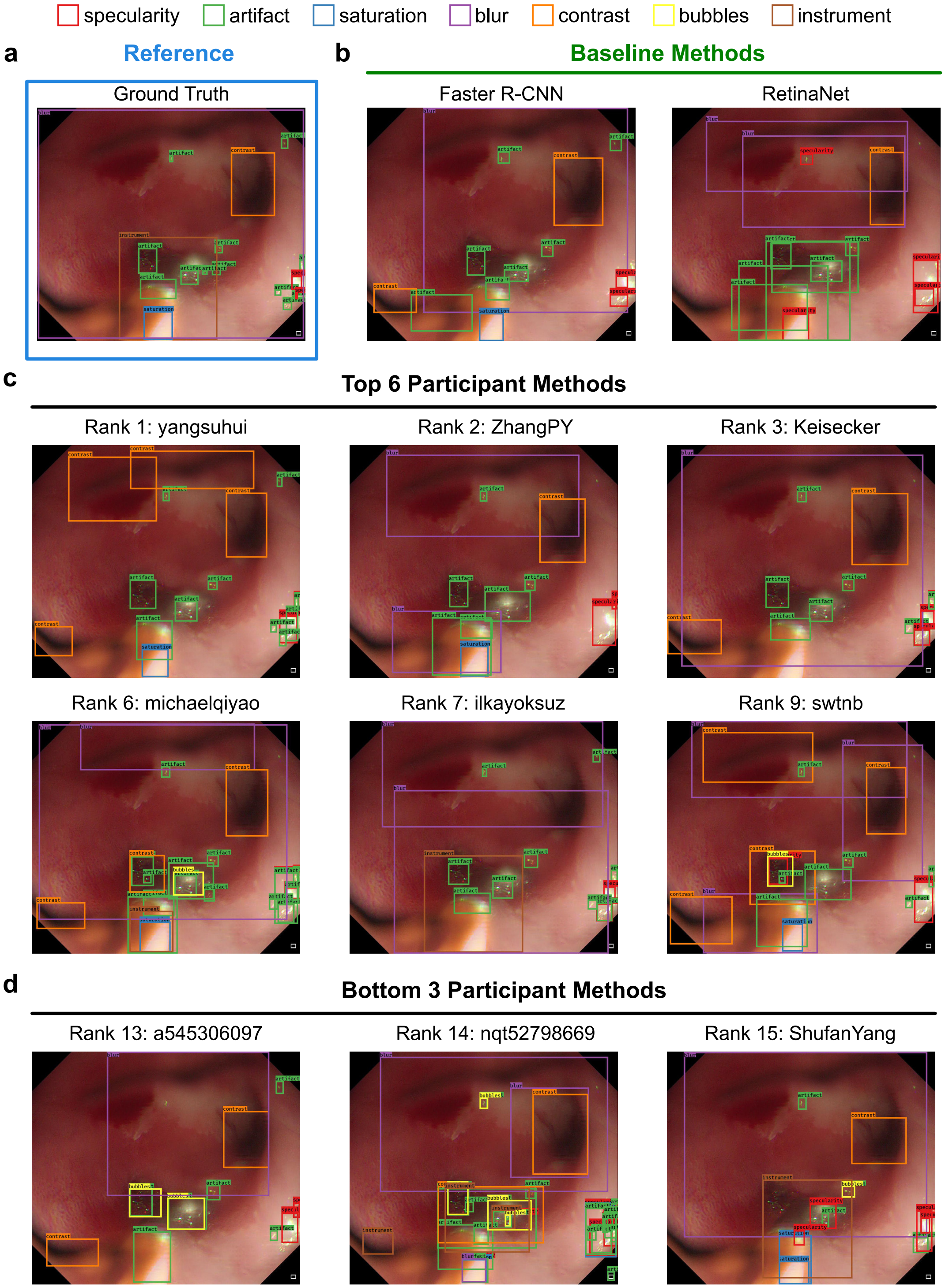

Figure 10. Example detected artifact bounding boxes. Detected boxes of ground-truth reference boxes generated by manual annotation according to protocols defined in **Supplementary Note I**, **a**), state-of-the art baseline methods, **b**), six top-ranking, **c**) and three bottom-ranking, **d**) participant detection methods.

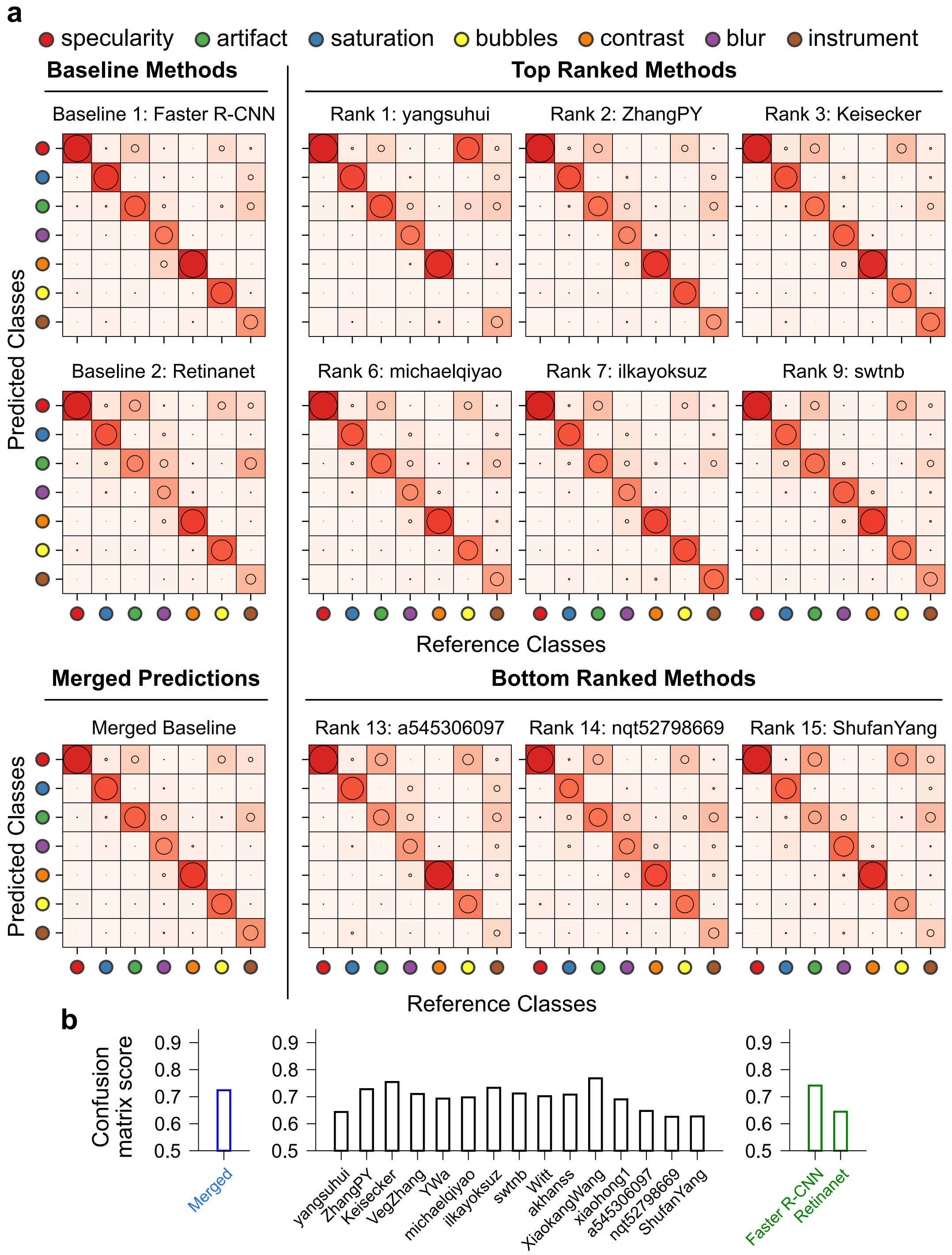

Figure 11. Confusion matrix assessment of baseline and selected EAD2019 participant artifact detection methods. **a**, Mean confusion matrices for individual baseline, top- and bottom- ranked submitted methods. The greater the values (0-1) the larger the circle and the redder the colour. For zero misclassification, the matrix should only be red, with circles only on the leading diagonal. **b**, Barplot of confusion matrix score (mean of matrix diagonal, **supplementary Note II**) for all individual baseline and participant detection methods.

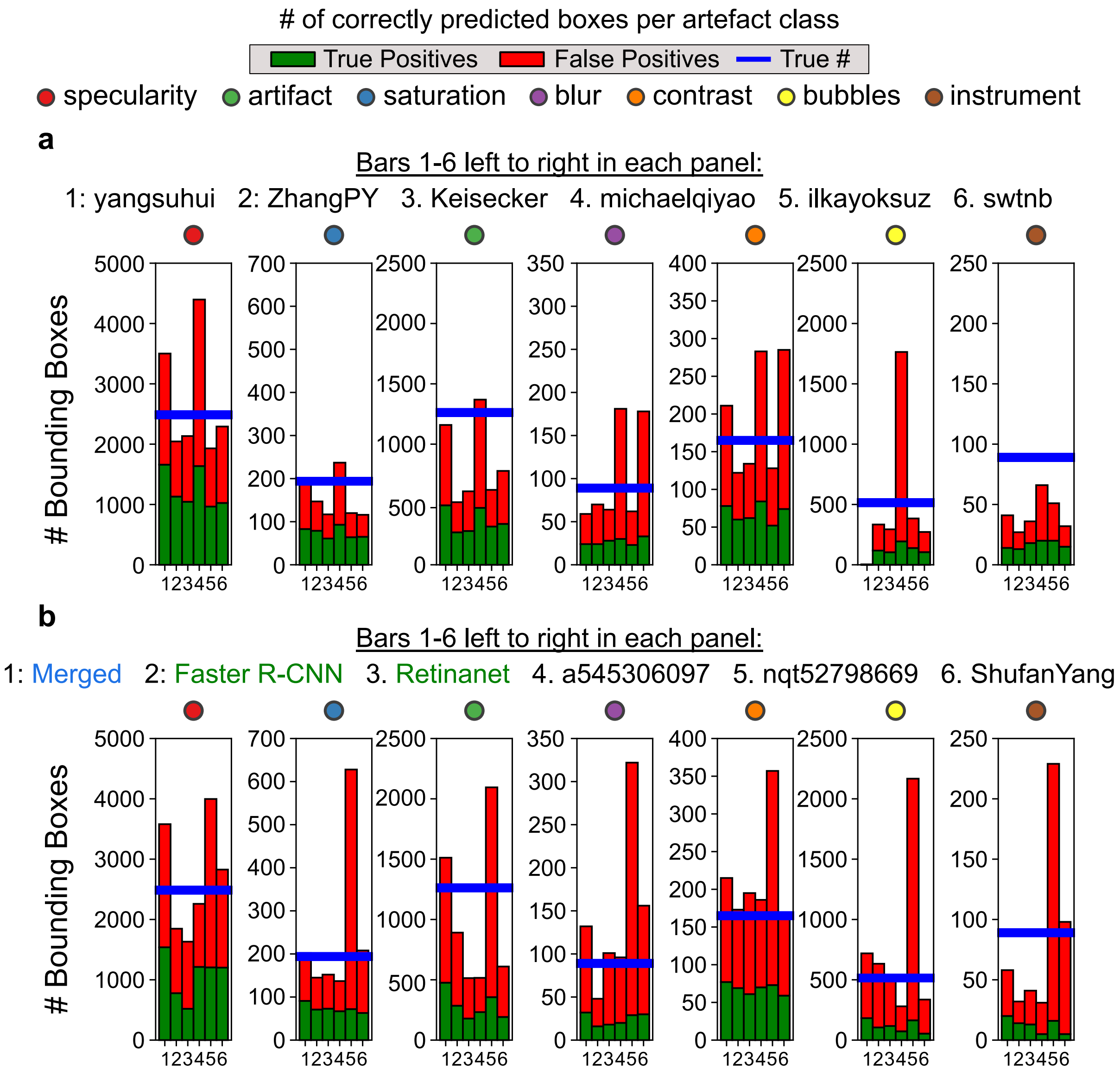

Figure 12. Number of predicted detection boxes per artefact class. Number of positive (IoU≥0.25) and negative (IoU<0.25) predicted bounding boxes for top ranking six participants, **a**), baseline and bottom ranking three participants, **b**). The number of ground-truth reference boxes per class is co-plotted as a thick horizontal blue line.

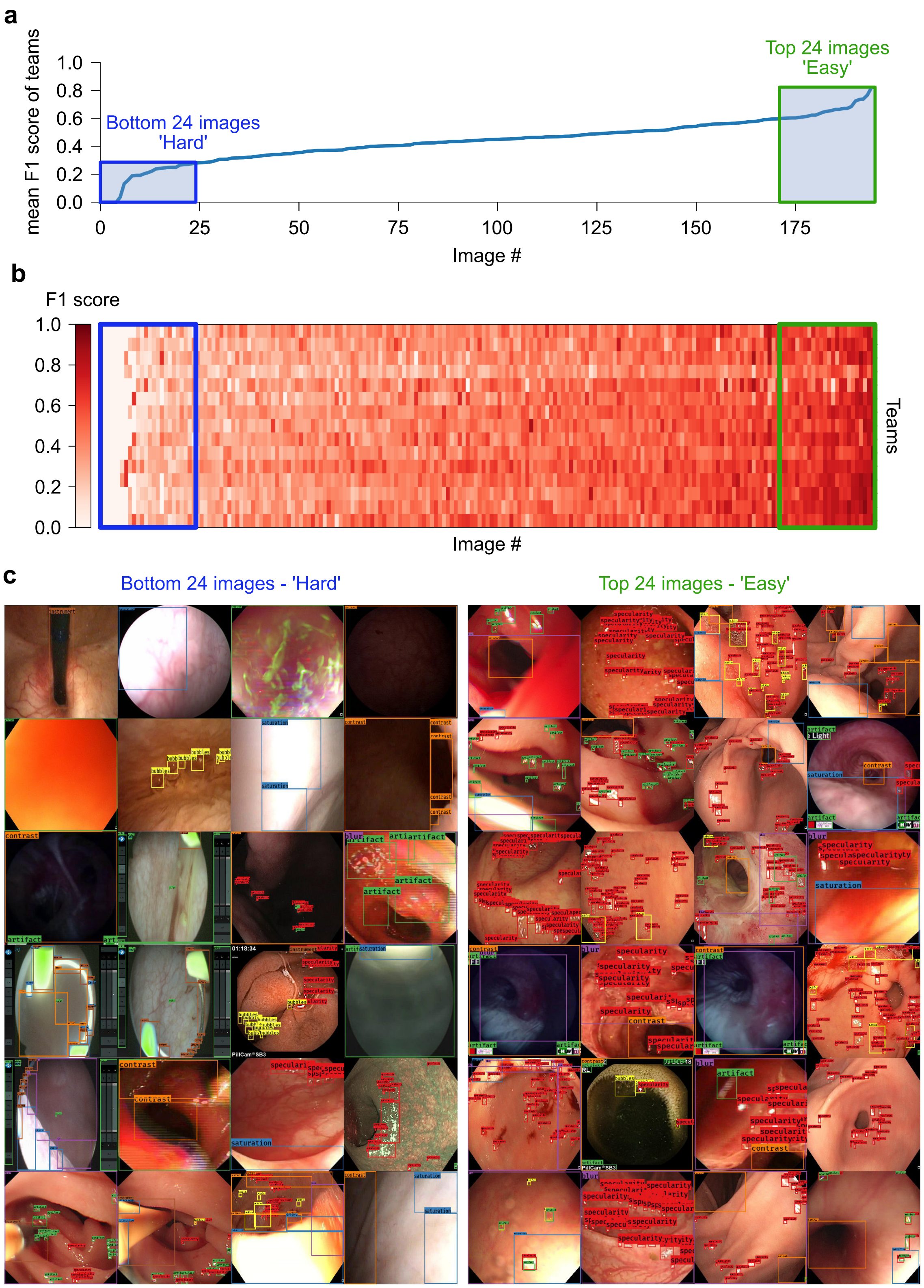

Figure 13. Globally easy and hard images for artefact detection. Ease of artefact detection for a given image in the detection test dataset was defined as the mean F1 score across all teams (**Supplementary Note II**). **a**, F1 score for each test image sorted by ascending mean F1 score. The higher the F1 score, the easier it was for all teams to locate all artefacts present in the image. **b**, Breakdown of F1 score per team (*y*-axis) for each test image in ascending mean F1 score order as in **a**). Teams were ordered top to bottom by descending detection score. **c**, Montage of the 24 hardest and easiest images for artefact detection. Images in the montages have been rescaled to 256 x 256 pixels for display.

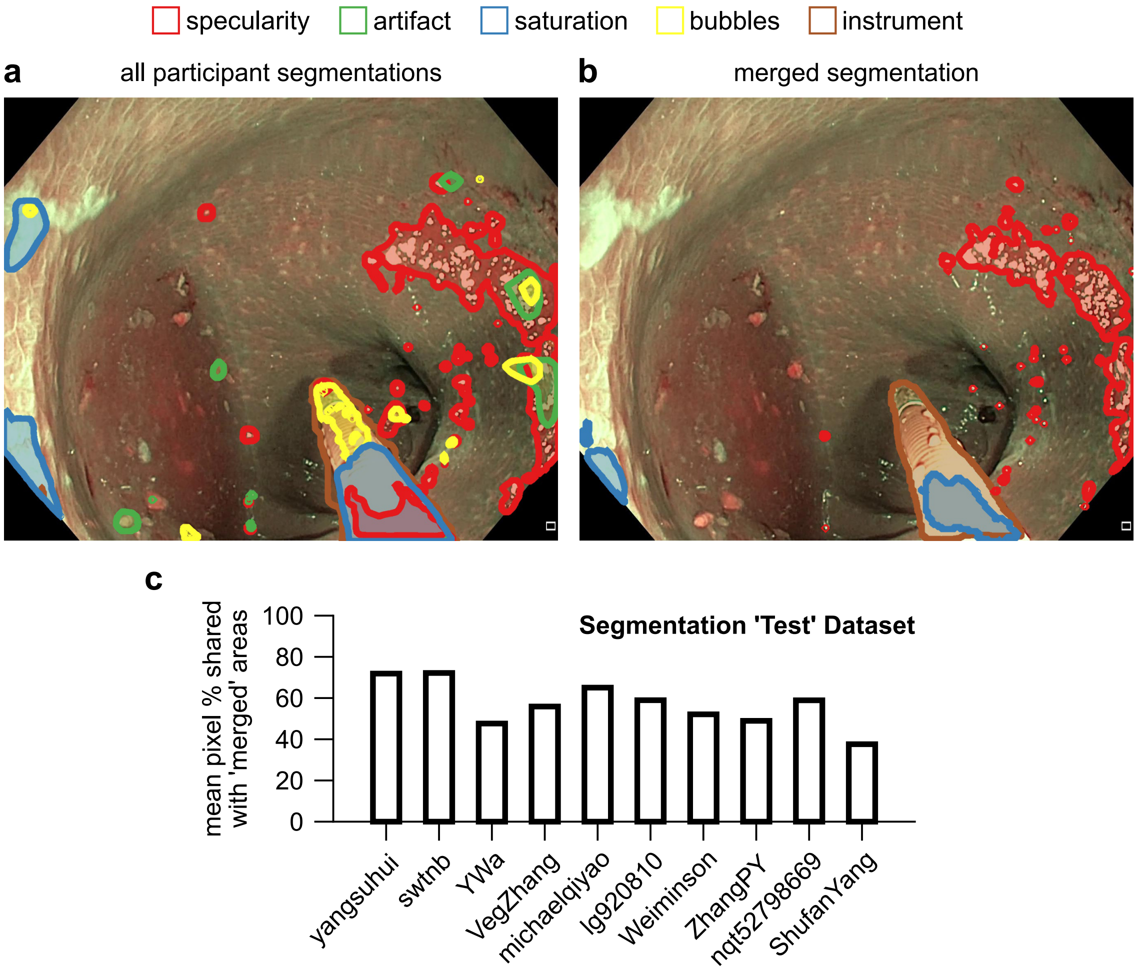

Figure 14. Super segmentation by retaining consensus segmented regions from all teams. **a** Overlay of all segmented regions from each of the 10 teams in the challenge on an example test image. **b**, Final merged predicted segmentation after retaining the stably segmented regions by consensus between teams. **c**, Mean area overlap between the final merged segmentation and individual team segmentations as the mean fraction of identically predicted pixels relative to the number of positively predicted pixels in the merged segmentation for all images in the segmentation test dataset (n = 121 images).

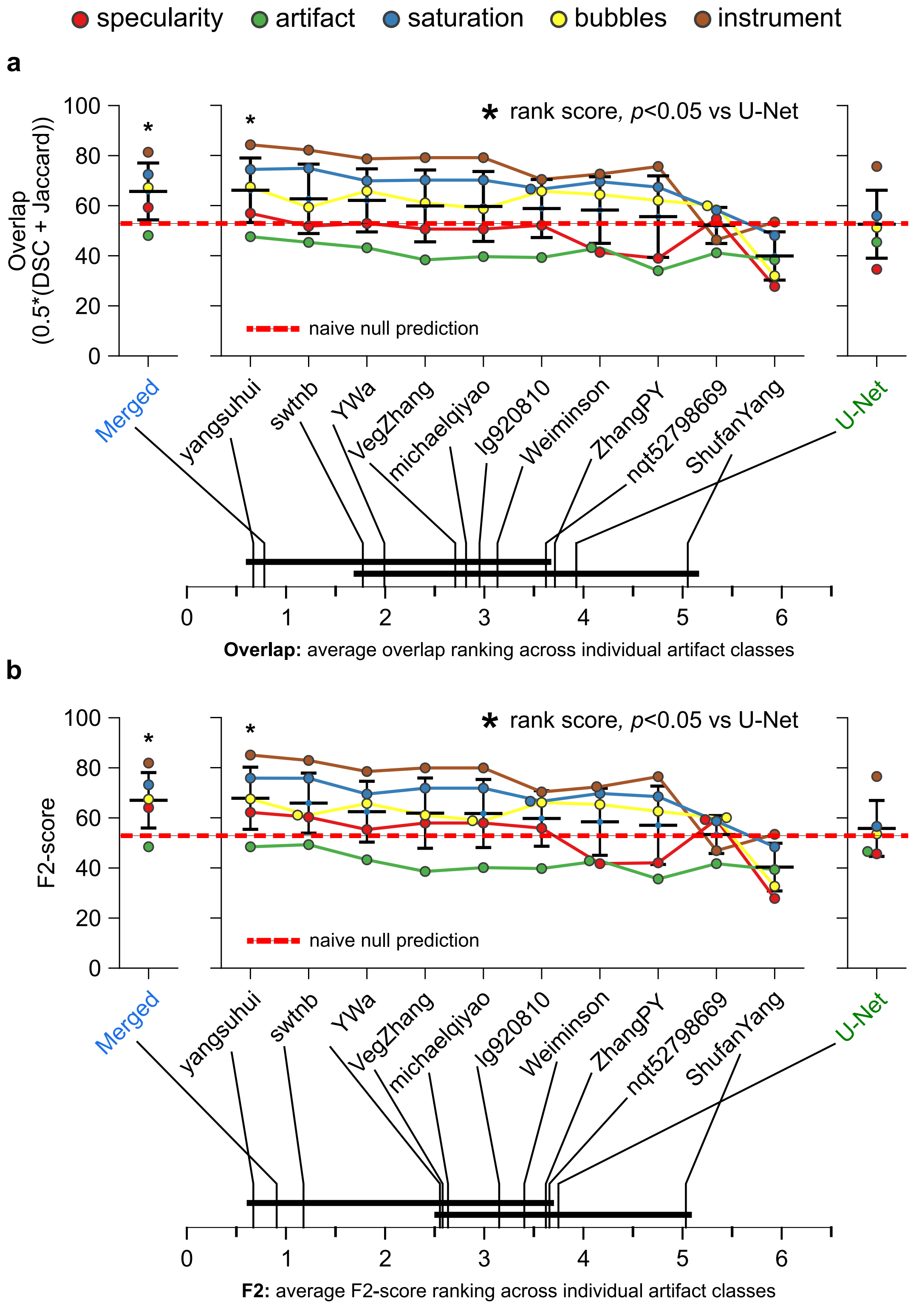

Figure 15. Statistical assessement of relative rank performance between teams with respect to overlap and F2 scores. Error and swarm plot of individual artefact class (top) and average rank performance (bottom) of individual teams over individual artefact classes for overlap **a**), and F2 **b**) scores. In all panels error bars plot ± 1 standard deviation of class scores relative to the global mean score per team and the red dashed line plots the score for a method which predicts negative for all classes for all images. '*' mark teams whose rank performance significantly improve on the U-Net baseline with Friedman Bonferroni-Dunn post-hoc testing and $p<0.05$. Solid black lines join methods with no significant difference in rank with Friedman-Nemenyi post-hoc testing and $p<0.05$.

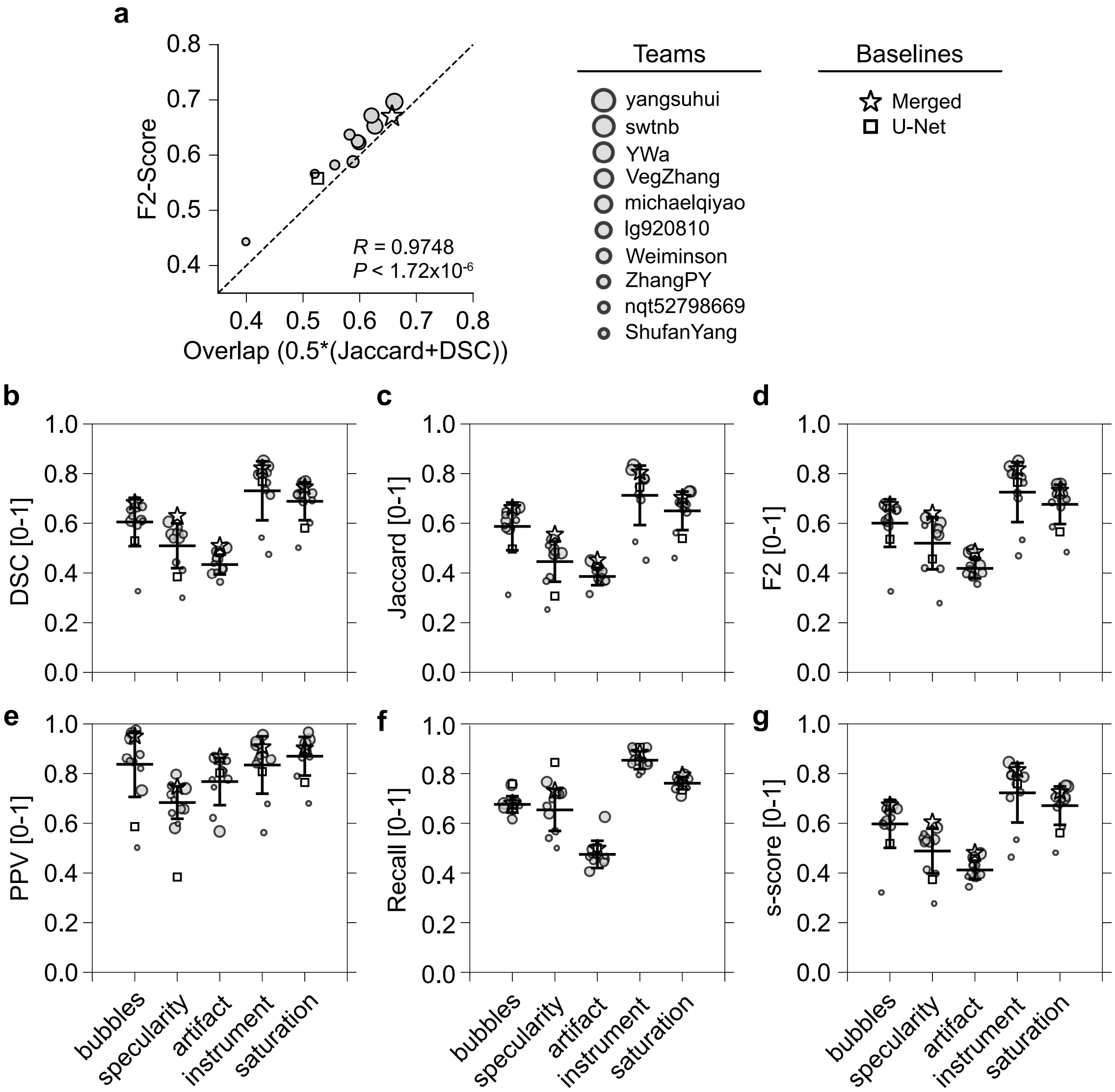

Figure 16. Class-specific segmentation performance with respect to different segmentation metrics. **a**, Plot of overlap vs F2 score. Dashed black line plot the identity line. Error bar plots of class-specific performance with respect to DSC, **b**), Jaccard, **c**), F2, **d**), PPV, **e**), recall, **f**) and segmentation or s-score, **g**) across all 10 teams. In panels **b**)-**g**) error bars plot $\pm$ 1 standard deviation of individual team scores relative to the mean team score with respect to the artefact class. In all panels teams are plotted with circle markers with size in decreasing size by decreasing mean s-scores.

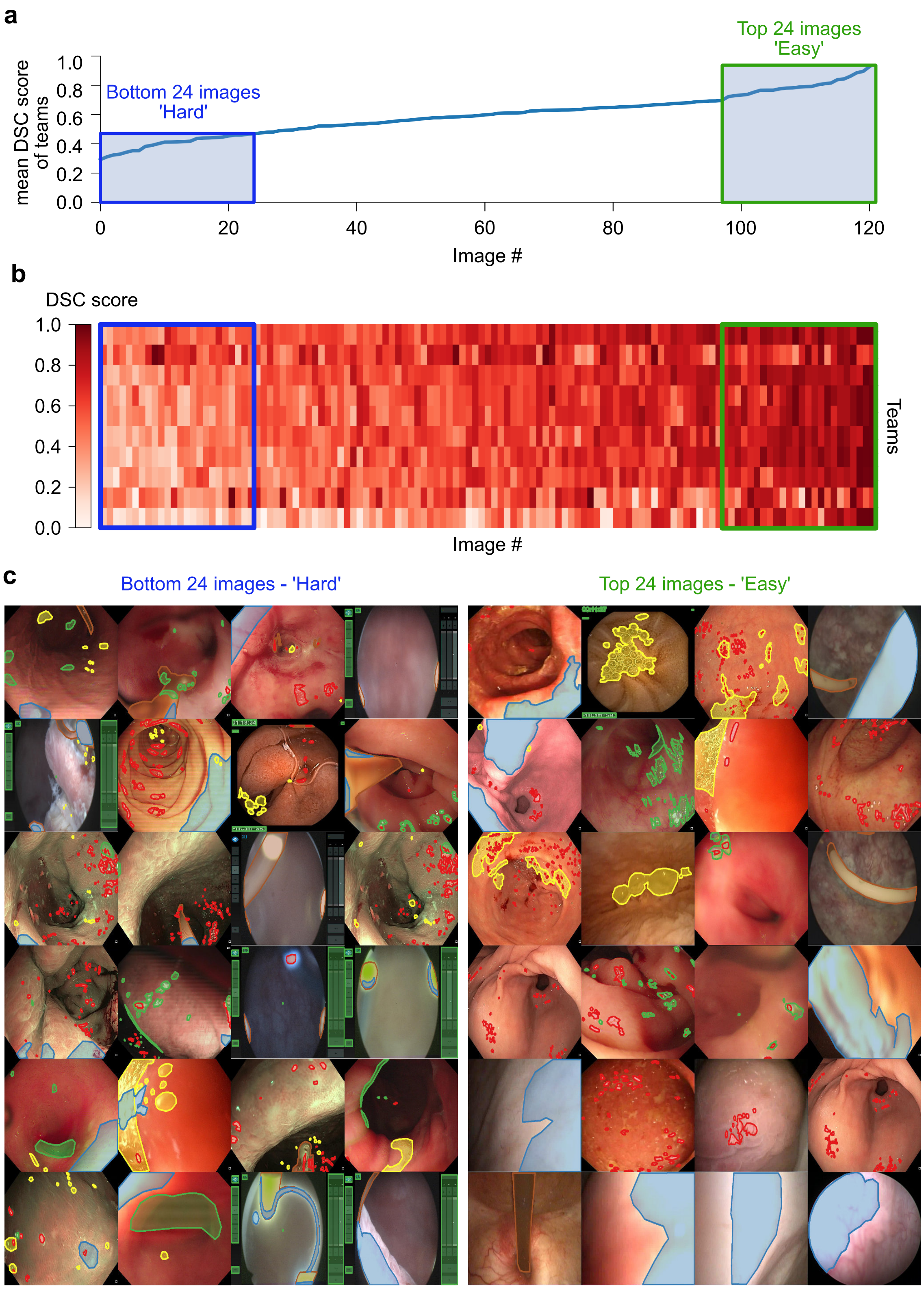

Figure 17. Globally easy and hard images for artefact segmentation. Ease of artefact detection for a given image in the segmentation test dataset was defined by the mean DSC score across all teams (**Supplementary Note II**). **a**, DSC score for each test image sorted by ascending mean DSC score. The higher the DSC score, the easier it was for all teams to segment all artefacts present in the image. **b**, Breakdown of DSC score per team (*y*-axis) for each test image in ascending mean F1 score order (*x*-axis) as in **a**). Teams were ordered top to bottom with descending segmentation score. **c**, Montage of the 24 hardest and easiest images to segment. Images in the montages have been rescaled to 256 x 256 pixels for display.

Supplementary Note I: EAD dataset

With the EAD Challenge we aimed to establish a first large and comprehensive dataset for "Endoscopy artefact detection" (see Suppl. Fig.1-3). The provided data was assembled from 6 different centers worldwide: John Radcliffe Hospital, Oxford, UK; ICL Cancer Institute, Nancy, France; Ambroise Parè Hospital of Boulogne-Billancourt, Paris, France; Instituto Oncologico Veneto, Padova, Italy; University Hospital Vaudois, Lausanne, Switzerland and the Botkin Clinical City Hospital, Moscow. This unique endoscopic video frame dataset is multi-organ (gastroscopy (stomach), cystoscopy bladder), gastrooesophageal (oesophagus), colonoscopy (colon), capsule endoscopy (small intestine)), multi-modal (white light, fluorescence, capsule and narrow band imaging), is inter patient and encompasses multiple populations (UK, France, Russia, and Switzerland). Videos were collected from patients on a first-come-first-served basis at Oxford, with randomized sampling at French centres and only cancer patients were selected at the Moscow centre. Videos at these centres were acquired with standard imaging protocols using endoscopes built by different companies; Olympus (Oxford, Paris), Biospec (Moscow), Medtronic (Oxford) and Karl Storz (EPFL, ICL, Paris). The dataset was built randomly mixing the collected data proportionately. Suppl. Fig. 3 gives a comprehensive visual breakdown of the dataset. All images have been carefully anonymised before release. No patient information should be visible in this data. We have also developed a comprehensive open-source software[1] to assist all participants.

Fabrication of dataset

Gold standard Clinical relevance to the challenge problem was first identified. During this step, 7 different common imaging artefact types (see Suppl. Fig. 3b) were suggested by 2 expert clinicians who performed bounding box labelling of these artefacts on a small dataset (∼100 frames). These frames were then taken as reference to produce bounding box annotations for the remaining train-test dataset by 2 experienced postdoctoral fellows. A final further validation by 2 experts (clinical endoscopists) was carried out to ensure the reference standard. The ground-truth labels were randomly sampled (1 per 20 frames) during this process. To maximize consistency in annotation between two independent annotators, a few rules were determined as described below:

Annotation software We used the open-source VIA annotation tool[2] for semantic segmentation. For bounding box annotation we used a python, Qt and OpenCV based in-house tool.

Annotation Protocols

- For the same region, multiple boxes were annotated if the region belonged to more than 1 class
- The minimal box sizes were used to describe the artefact region, e.g. if multiple specular reflections are present in an image then instead of one large box we use multiple small boxes to capture the natural size of the artefact
- Each artefact type was determined to be distinctive and general across endoscopy datasets

Annotator Variation

- Variation in bounding box annotations were accounted for by computing a weighted final score 0.6*mAP + 0.4*IoU in the multi-class artefact detection challenge. Here, IoU (intersection over union) was downweighted as it is likely to vary more compared to mAP (mean average precision) across individual annotators.
- Variation in the semantic class labels of masks for semantic segmentation was found not significant. Further we do not consider contrast and blur classes which are inherently poorly defined spatially..

Composition for different sub-challenges

Below we describe the composition of the training-test dataset for each sub-tasks of the EAD challenge. The information is also visually represented in Suppl. Fig.3:

Detection The training dataset for detection consists in total 2192 annotated frames over all 7 artefact classes. All algorithms were evaluated online[2] using the evaluation metrics discussed in **Supplementary Note II** on a test set of 195 frames (∼10% of training data). During the annotation we found that most of frames were much more affected by specularity, imaging artefact and bubbles compared to other artefact classes. We tried to keep the ratio of class types similar between the training and test datasets as best we could. Suppl.Fig.3 shows the artefact class distribution for detection and generalization datasets.

[1] Useful tools for this dataset: https://sharibox.github.io/EAD2019/

[2] https://ead2019.grand-challenge.org/evaluation/results/

Semantic Segmentation The training dataset for semantic segmentation consists 475 annotated frames for 5 of the 7 classes: specularity, saturation, artefact, bubbles and instrument (i.e., no contrast and blur). The test data contains 122 annotated frames.

Generalization The training dataset for generalization is the same as that for detection however the test data for generalization uses a previously withheld dataset provided by a sixth institution (Padova) not present in any other training or test data released for the detection and segmentation tasks (Suppl.Fig.3b). The generalization test data consists 52 images and the task was to detect all 7 artefact classes as with the detection task.

Image variation within dataset

We highlight in this section the diversity of the assembled dataset in terms of visual appearance and variability in artefacts.

Image modality Imaging modality plays an important role in visual diversity of clinical endoscopy data. Different imaging modalities are commonly used during diagnosis to better visualize the underlying disease which inevitably changes the appearance of the imaged tissue of an organ.

- **White light (WL)** WL is considered as an standard imaging modality. Broad spectrum light is shone and all reflected wavelengths are collected to form a true to life representation of the tissue surface (mucosa). The captured visual appearance is what the human eye normally sees; pink tissue, low contrast with surface vasculature. Unfortunately, due to multi-focal nature of the tumors, the specificity of such imaging modality is very low.

- **Fluorescence light (FL)** FL is used as a complimentary modality in addition to WL due to its improved specificity for detecting cancer tumors in hollow organs. During FL, a colored dye is introduced which selectively visualizes targeted tissue regions at different color and wavelength. Common application include the identification of multi-focal bladder cancer, squamous cell carcinomas and dysplasia in oesophagus and screening for dysplasia in individuals with ulcerative colitis[3].

- **Narrow band imaging (NBI)** is an imaging technique that uses specific blue and green wavelengths to enhance the detail of the tissue surface. This is to target the peak light absorption of haemoglobin in the blood. Consequently, blood vessels appear darker and improves visual contrast for the identification of other surface structures[4]. It is frequently used to aid identification of Barrett's oesopagus[5] and pit patterns for colorectal polyps and tumour classification[6]. The research have shown its improved accuracy and specificity compared to WL modality.

- **Capsule imaging** is a procedure that uses a much smaller wireless camera inside a vitamin-size capsule which the patient swallows to capture images compared to the standard flexible tube. It is commonly used to image the small intestine, an area which otherwise would be difficult to access. Strictly not an imaging modality but a type of instrumentation we chose to include it as a modality as the captured images of the small intestine using the smaller camera form factor are distinctively different from the above three modalities. Further the imaging in water of the PillCam in the EAD dataset results in bubbles of much larger areas.

Imaged organs

- **Oesophagus.** The oesophagus is a hollow tube that connects the mouth with the stomach. Two major cancers occur here; squamous cell carinoma in the upper and middle oesophagus near the mouth and adenocarcioma in the lower oesophagus at the junction with the stomach. A premalignant lesion that can precede adenocarcinoma is call Barrett's Oesophagus. The surface appearance of the oesophagus is smooth ending in a sphincter leading towards the stomach. This transition is characterised visually by smooth tissue giving way to the rougher, glandular looking stomach tissue. Common conditions include cancer, oesophagitis (inflammation) and Barrett's oesophagus.

- **Stomach.** The stomach is a hollow organ that receives food from the oesophagus. Visually the stomach is most distinguished by its glandular, rough tissue and under white light, presence of the two sphincters, one to the oesophagus, one to the lower intestine. Conditions include gastritis and cancer.

- **Small Intestine.** The small intestine absorbs nutrient from food. Of the organs listed, it is the most difficult to reach, endoscopy of oesophagus and stomach through the mouth, colon endoscopy through anus and bladder endoscopy stomach through the urethra. Under capsule endoscopy (PillCam) the surface is tentacle-like with many villi. Conditions include Crohn's disease, irritable bowel syndrome and cancer.

- **Colon.** The colon, also called the large bowel or intestine removes water and salt to form stool. Endoscopy of the colon is called colonoscopy. Common conditions include colitis (inflammation of the colon) and colon cancer. The appearance of the colon is tube-like with regular surface undulations.

- **Bladder.** The bladder is a muscular sac in the pelvis, above and behind the pubic bone. The bladder stores urine. Endoscopy of the bladder is called cystoscopy. It is used to assess conditions such as cystitis (inflammation of the bladder) and bladder cancer. The surface appearance of the bladder looks typically smooth with lots of microvasculature.

Imaging instrument The imaging instrument used depends on what is being imaged which often determines the specialist manufacturer. In our dataset endoscopy images of the oesophagus (Oxford), stomach (Oxford, Paris, Padova) and colon (Oxford, Padova) uses Olympus, bladder (EPFL, ICL, Moscow) uses Karl Storz and the small intestine (Oxford) uses Medtronic's PillCAM SB3 system.

Visualisation of image diversity using t-SNE To capture the image diversity based on texture and colour we trained a ResNet 'encoder-decoder' autoencoder to extract deep image features for t-SNE (Table 1,2). All images were rescaled by bilinear interpolation to be 256 x 256. ResNet blocks use the full pre-activation variant[7]. The autoencoder was trained on the detection training set with mean absolute error loss and Adam optimizer (λ=1x10^4, β_1=0.5, β_2=0.99) with early stopping and no data augmentation. The output of the dense layer in the encoder (Table 1) was used as input to t-SNE (perplexity = 15, learning rate = 100) to reduce the 256 dimensions to 2 dimensions for plotting, (Suppl.Fig. 2). By visual inspection, individual images are well-spread in all directions with some of the generalization dataset images occupying new areas not occupied by train and test datasets in the t-SNE embedding. This indicates diversity. Further we see the detection test dataset is representative of the training dataset with images embedded in the areas covered by both 'Train-I' and 'Train-II'.

Layer	Filters	Strides	Size
Conv2D-BN-ReLU	3x3x16	1	256 x 256 x 16
Resblock x 2	3x3x16	1	256 x 256 x 16
Conv2D-BN-ReLU	2x2x32	2	128 x 128 x 32
Resblock x 2	3x3x32	1	128 x 128 x 32
Conv2D-BN-ReLU	2x2x32	2	64 x 64 x 32
Resblock x 2	3x3x32	1	64 x 64 x 32
Conv2D-BN-ReLU	2x2x64	2	32 x 32 x 64
Resblock x 2	3x3x64	1	32 x 32 x 64
Conv2D-BN-ReLU	2x2x64	2	16 x 16 x 64
Resblock x 2	3x3x64	1	16 x 16 x 64
Conv2D-BN-ReLU	2x2x128	2	8 x 8 x 128
Resblock x 2	3x3x128	1	8 x 8 x 128
Flatten	-	-	8192
Dense	256	-	256

Table 1. Architecture of encoder network in ResNet autoencoder.

Layer	Filters	Strides	Size
Dense	8192	-	8192
Reshape	-	-	8 x 8 x 128
UpSample	-	2	16 x 16 x 128
Conv2D-BN-ReLU	3x3x64	1	16 x 16 x 64
UpSample	-	2	32 x 32 x 64
Conv2D-BN-ReLU	3x3x32	1	32 x 32 x 32
UpSample	-	2	64 x 64 x 32
Conv2D-BN-ReLU	3x3x32	1	64 x 64 x 32
UpSample	-	2	128 x 128 x 32
Conv2D-BN-ReLU	3x3x16	1	128 x 128 x 16
UpSample	-	2	256 x 256 x 16
Conv2D-ReLU	3x3x3	1	256 x 256 x 3
Conv2D-Sigmoid	1x1x3	1	256 x 256 x 3

Table 2. Architecture of decoder network in ResNet autoencoder.

Image Quality Metric	Train	Test	Generalization
LAPV	3790±2125	3701±1471	3738±2386
BRISQUE	42.27±14.35	58.05±23.29	48.61±18.84
Visual diversity index	0.85	0.69	0.81
% artefact area	47± 29	55±34	48±28
% overlapped boxes (IoU>0)	51±40	65±32	57±27
Artefact (Shannon) diversity index	0.72±0.41	0.73±0.38	0.96±0.30
Artefact (Simpson's) diversity index	0.42±0.23	0.41±0.21	0.53±0.14

Table 3. Computed image quality metrics for the EAD dataset. LAPV was computed with a 5x5 Laplacian kernel. IoU - measure of spatial overlap using intersection-over-union, see **Supplementary Note II**.

Image quality measures To provide more objective quantification of the variability in endoscopic image a number of image quality measures were computed in Table 3. Without corresponding 'clean' reference images, standard full-reference image quality measures such as PSNR and SSIM could not be used. Instead we used no-reference image quality measures from the literature which do not require matched 'clean' versions of the image; the Variance of Laplacian (LAPV)[8] to quantify the amount of edges in an image (primarily useful for detecting image blur) and BRISQUE[9] which quantifies the extent of visual distortion (thus theoretically independent of artefact type). These metrics have have primarily been developed and tuned on natural images which have very different image statistics compared to biomedical endoscopy images; more diverse colours, distinguished edges and distinctive image texture. As such we computed the following additional estimates of quality based on the trained autoencoder featueres and manually labelled endoscopy artefact bounding box annotations.

- **Visual diversity index** We compute the ratio of the explained variance of the second and first principal component, σ_2/σ_2 of applying PCA analysis using the extracted features of the trained deep autoencoder. The larger the diversity the smaller the variation that can be captured by the first component relative to the second component.

- **% Artefact area** The average % of the image area occupied by imaging artefact. Measures how much of the image is affected by imaging artefact.

- **% overlapped boxes** The % of bounding boxes that overlap another bounding box of any class. Measures the spatial proximity of individual artefacts.

- **Artefact diversity indices** Measures the variation in artefact types in an image. The more class types in an image, the higher the expected image appearance variation. We use two common quantitative measures used to measure ecological diversity; Shannon and Simpson's diversity indices. For both, the higher the measure the greater the diversity, the more types of artefact that is present per image. If all 7 artefact classes are present we expect Shannon and Simpson's diversity index values of 1.95 and 0.86 respectively.

  - *Shannon diversity index (H).* More commonly known as information entropy in information sciences. It quantifies the uncertainty in predicting the artefact class of an individual bounding box taken at random from the dataset.

    $$H = -\sum_{i=1}^{N} p_i \ln p_i$$

    where p_i is the proportion of bounding boxes belonging to the ith of N artefact class in the dataset of interest.

  - *Simpson diversity index (D).* Measures 1 - the probability that two bounding boxes randomly selected from the same dataset belong to the same artefact class without replacement.

    $$D = 1 - \sum_{i=1}^{N} \frac{n_i(n_i-1)}{N(N-1)}$$

    where n_i is the number of bounding boxes in the ith of N artefact classes in the dataset of interest. Simpson's diversity index gives a value 0-1 for increasing diversity (more class types).

Frequency of images with overlap of bounding boxes between different artefact classes. A major problem of endoscopy imaging artefacts compared to the detection of objects in natural images is the potential for numerous spatial overlapping between artefact bounding boxes. Intuitively this will complicate accurate artefact detection. To better understand the frequency of spatial overlap generally in endoscopy image datasets, we further dissected the % overlapped boxes between artefact classes (Table 3) with respect to individual artefact classes and frequency across an image dataset. For a given dataset, $\mathscr{D}$ of N images three measures was used.

- *The frequency (%) of images with box overlap between artefact class i and j*, (Suppl.Fig.4a). The fraction of the total number of images containing a box of class i that overlap with a box of class j. Boxes were deemed to overlap if IoU>0.

$$\{\text{Frequency overlap } (\%)\}_{ij}^{\mathscr{D}} = \frac{1}{N}\sum_{n=1}^{N} \mathbb{I}(\text{IoU}_{ij} > 0)$$

where $\mathbb{I}$ denotes the indicator function for counting.

- *The proportion (%) of overlapped boxes in artefact class i that overlapped with a overlapped box in class j*, (Suppl.Fig.4b). This is essentially the row normalised matrix of the frequency overlap measure above.

$$\{\text{Cross class overlap } (\%)\}_{ij}^{\mathscr{D}} = \frac{\{\text{Frequency overlap } (\%)\}_{ij}}{\sum_{\forall j}\{\text{Frequency overlap } (\%)\}_{ij}}$$

- *Mean IoU of overlapped boxes in class i with overlapped boxes in class j*, (Suppl.Fig.4c).

$$\{\text{Mean IoU } (\%)\}_{ij}^{\mathscr{D}} = \frac{1}{N}\sum_{n=1}^{N} \text{IoU}_{ij}$$

Supplementary Note II: Evaluation methods

Performance Criteria (technical measures used in the challenge)

Intersection over union (IoU) and Jaccard index (J). We quantify the amount of overlap between reference and predicted bounding boxes and segmentations using the intersection-over-union, (Suppl.Fig.5a). defined as

$$\text{IoU}(R,S) \text{ or } \text{J}(R,S) = \frac{|R \cap S|}{|R \cup S|} \tag{1}$$

where R is the reference bounding box/segmentation of an artefact and S its corresponding predicted bounding box/segmentation and $|\cdot|$ denote the set cardinality. The IoU is more commonly known as the Jaccard index in image segmentation. For multiple artefact classes the mean IoU/Jaccard index calculated over all reference bounding boxes in the dataset images is used for evaluation. The IoU ranges between 0 for no overlap to 1 for perfect overlap.

Mean average precision (mAP). For any given image the number of predicted bounding boxes typically will not equal the number of reference bounding boxes. Intuitively, the best algorithm is the one that minimises the total number (#) of predictions or precision $p = \frac{TP}{TP+FP}$ and maximises the total number of detected reference boxes or recall, $r = \frac{TP}{TP+FN}$. The average precision (AP) is the standard measure used to evaluate this tradeoff for object detectors taking into account the detector's confidence of a box containing an object (objectness score). Average precision (AP) is computed as the Area Under Curve (AUC) of the precision-recall curve of detection sampled at all unique recall values $(r_1, r_2, ...)$ whenever the maximum precision value drops:

$$\text{AP} = \sum_n \left\{ (r_{n+1} - r_n)\, p_{\text{interp}}(r_{n+1}) \right\}, \tag{2}$$

with $p_{\text{interp}}(r_{n+1}) = \max_{\tilde{r} \geq r_{n+1}} p(\tilde{r})$. Here, $p(r_n)$ denotes the precision value at a given recall value. This definition ensures a monotonically decreasing precision curve. The mAP is the mean of AP over all artefact classes i for $N = 7$ classes given as

$$\text{mAP} = \frac{1}{N} \sum_i \text{AP}_i \tag{3}$$

This definition of AP was popularised in the PASCAL VOC challenge[10]. The calculation is illustrated in Suppl.Fig.5B,C. Considering variation in annotation, we used an IoU>= 0.25 to designate a positive "match" between a reference and predicted box. The mAP ranges between 0 for no detection and 1 for full detection. The higher the mAP the better the performance.

Artefact detection accuracy (score_d). The detection performance of participants were finally ranked using a weighted score of 0.6 mAP + 0.4 IoU.

Dice coefficient (DSC). A spatial overlap measure for segmentation similar to IoU defined as $\text{DSC}(R,S) = \frac{2|R \cap S|}{|R|+|S|}$ where $|\cdot|$ denotes the set cardinality and R and S is the reference and predicted masks respectively. DSC is 0 for no overlap and 1 for perfect overlap. It can be calculated from the IoU, $\text{DSC} = \frac{2\text{IoU}}{1+\text{IoU}}$.

Precision (p), recall (r) and F_β score. These measures are used to evaluate the fraction of correctly predicted instances. Given a number (#GT) of true instances (ground-truth bounding boxes or pixels in image segmentation) and a method which predicts #Pred instances, precision is the fraction of predicted instances that were correctly found, $p = \frac{\#\text{TP}}{\#\text{Pred.}}$ where TP denotes true positive and recall is the fraction of ground-truth instances that were correctly predicted, $r = \frac{\#\text{TP}}{\#\text{GT}}$. Ideally, the best methods should have jointly high precision and recall. F_β-scores gives a single score to capture this desirability through a weighted (β) harmonic means of precision and recall, $\text{F}_\beta = (1+\beta^2) \cdot \frac{p \cdot r}{(\beta^2 \cdot p)+r}$.

Segmentation accuracy (score_s or s-score). Semantic segmentation accuracy was measured with similar consideration to that of detection accuracy using a combined weighted score,

$$\text{score}_\text{s} \text{ or s-score} = 0.75 \cdot [0.5 \cdot (\text{F}_1 + \text{J})] + 0.25 \cdot \text{F}_2$$

taking into account the overlap between predicted and reference segmentation as given by the Jaccard Index and the precision-recall tradeoff as given by the DSC similarity coefficient (DSC) also called F_1-score and the F_2-score.

Generalization score. We define generalization of artefact detection as the stability of an algorithm to achieve similar performance when applied to a different imaging dataset that may differ in imaging modality and acquisition protocol but contain the same imaging artefact classes. To assess this, participants applied their trained methods to data collected from a sixth institution whose images were not included in neither the training nor test data of the detection and segmentation tasks. Without access to the training code, we estimated the generalization ability as the mean deviation between the mAP of the detection and generalization test datasets of each class i for deviation greater than a tolerance of 0.1 $\mathrm{mAP}_{\mathrm{d}}^{i}$.

$$\mathrm{dev_g} = \frac{1}{N}\sum_i \mathrm{dev_g}^i \tag{4}$$

$$\mathrm{dev_g}^i = \begin{cases} 0, & \text{for } |\mathrm{mAP_d}^i - \mathrm{mAP_g}^i|/\mathrm{mAP_d}^i \leq 0.1 \\ |\mathrm{mAP_d}^i - \mathrm{mAP_g}^i|, & \text{for } |\mathrm{mAP_d}^i - \mathrm{mAP_g}^i|/\mathrm{mAP_d}^i > 0.1 \end{cases} \tag{5}$$

The best algorithm should have high $\mathrm{mAP_g}$ and low $\mathrm{dev_g}(\rightarrow 0)$. In practice, participants were finally ranked using a weighted ranking score, $\mathrm{score_g} = 1/3 \cdot \mathrm{Rank}(\mathrm{dev_g}) + 2/3 \cdot \mathrm{Rank}(\mathrm{mAP_g})$ where $\mathrm{Rank}(\mathrm{mAP_g})$ is the rank of a participant when sorted by $\mathrm{mAP_g}$ in ascending order.

Performance Criteria (technical measures for targeted analysis)

Building super detection by merging participant bounding box predictions. For each image, all bounding box predictions from all teams were concatenated together to form a super-set of box predictions, B. The super-set B was then filtered to produce the final "super detector" predictions:

1 **Stability of detection** – for each box in B, retain only boxes that overlap (IIoU $\geq$ 0.5, same artifact class) with boxes predicted by at least 5 teams. The boxes that remain forms a set B_{stable}

2 **Non-maximum suppression** – Standard bounding box non-maximum suppression post-processing (IoU $\geq$ 0.25) is then applied to all boxes in B_{stable} to compute the final detection.

Contribution of individual teams to performance of super detector We measure the contribution of individual teams to the super detector by the mean percentage of merged boxes per image in the given dataset taken from each team. Given two detectors that produce similar bounding boxes the detector that scores the most stable predicted bounding boxes with higher confidence is superior and will contribute most to the super detector. Suppl. Fig. 7c illustrates how not all the bounding boxes in the merged detector come from top-ranking methods.

Building super segmentation by merging participant segmentation masks. All predicted binary segmentation masks were summed together for each artifact class independently and divided by the number of teams to generate a consensus score, 0-1 between teams. Thus, a pixel in the merged mask prediction has value 1 only if all teams predicted the artifact class was present. The final merged mask is the result retains all predicted pixels if at least 4 teams predicted the class i.e. consensus score $>=$ 0.4. This is a threshold essentially on the level of consensus to retain only "stable" predictions.

Contribution of individual teams to performance of super segmentation We measure the contribution of individual team predictons to the super segmentation as the mean fraction of pixels shared between the individual team predictions and the super segmentation binary masks across artifact classes.

Confusion matrix of class detection. Measures the proportion (as a fraction 0-1) of the number of predicted boxes that were correctly classified. Predicted boxes for each image were ranked in descending order by predicted objectness score independent of class and assigned to the ground truth bounding box with highest IoU. For each class, the number of times its bounding boxes were assigned to each ground truth artefact class was then tabulated and normalized by the total number of ground truth bounding boxes in the class. Predicted boxes with no ground truth box were ignored and do not contribute to the computation. For an image dataset, the average confusion matrix over all images was reported. The ideal matrix has all leading diagonal elements 1 and all off-diagonal elements 0.

Confusion matrix score of class detection. To summarize the confusion matrix performance with a single matrix we report the mean of the trace of the confusion matrix (C_{ij}) of N artefact classes.

$$\text{Confusion matrix score} = \frac{\mathrm{Tr}(C_{ij})}{N}$$

The higher the score, the better the ability of a detection method to distinguish between artefact classes.

Positive predictive value (PPV) and recall for segmentation. This is the same as the definition of precision and recall respectively for artefact detection using individual pixels instead of bounding boxes.

Statistical correlation tests. Three different statistical tests was used. For all statistical tests, p-value < 0.05 was deemed a statistically significant result to reject the null hypothesis, H_0.

- *Test of linear correlation between two continuous variables*. Pearson's correlation coefficient was used. The null, H_0 and alternative, H_A hypotheses with at least one strict inequality are

$$H_0 : \text{No linear correlation}$$
$$H_A : \text{Evidence for linear correlation}$$

- *Test of correlation between two sets of rankings*. Spearman's rank correlation coefficient was used. The null, H_0 and alternative, H_A hypotheses with at least one strict inequality are

$$H_0 : \text{No evidence of correlated rank ordering}$$
$$H_A : \text{Evidence of correlated rank ordering}$$

- *Test for monotonic ordering of categorical variables*. The one-sided Jonckheere-Terpestra test [11,12] was used to test for statistical evidence of increasing ordering of algorithm performance across artefact classes. For k groups (where $k > 2$) the null, H_0 and alternative, H_A hypotheses with at least one strict inequality are

$$H_0 : \theta_1 = \theta_2 = \cdots = \theta_k$$
$$H_A : \theta_1 \leq \theta_2 \leq \cdots \leq \theta_k$$

  where θ_i is the population median of the i-th group. The null hypothesis was rejected for p value ≤ 0.05.

Statistical relative rank performance tests. To evaluate if a participant method statistically outperform other proposed methods in detection or segmentation, the non-parametric post-hoc Friedman-Nemenyi testing of Demšar et al.[1] was applied. Treating each artifact class as a separate dataset, the Friedman test is first applied to test if differences exist across the compared methods. Given a significant result, $(p < 0.05)$, the Nemenyi test is applied to find the groups of methods that differ by pairwise comparison of the mean rank sums from the Friedman test. The results of the pairwise significance testing is then visualised in the form of a critical difference diagram proposed by Demšar et al. where non-significantly different methods are joined together with a thick horizontal line (e.g. Suppl. Fig. 9). To compare individual methods to a given control method following a positive Friedman test to ascertain differences we applied Bonferroni-Dunn as a more powerful test as suggested by Demšar et al.[1] to test for statistical difference. The results of this test is visualised in the form of markers above the respective methods which show significant statistical difference, (e.g. Suppl. Fig. 9). To implement the statistical tests we used the Python-based library, STAC (http://tec.citius.usc.es/stac/doc/).

Performance Criteria (measures for practical applicability)

Real-time performance assessment. The primary metric of assessment is the test time and GPU (graphics card) used. The higher the compute capability of a GPU, the faster the performance. However this only holds for a single GPU. Multiple GPUs of lower compute capability can achieve faster performance than a single GPU of higher compute capability. In addition GPU's with higher memory enables training of large images at native resolution with more data augmentation which can significantly improve performance. Unfortunately, the inherently voluntary nature of participation prevents accurate determination and fair comparison across all submitted methods.

Clinical usability assessment. The critical factors for clinical application are i) accuracy, ii) consistency of prediction and iii) computational efficiency. Of the three, consistency is a crucial consideration. Given the ethical implications, out of two algorithms, the one that produces the least number false positives with least variability is more preferable for similar accuracy scores. Based on these considerations we thus computed the clinical relevance score of a method as a weighted average of the individual rankings of the the three factors with consistency the most weighted.

$$\text{Clinical relevance rank score} = w^{acc}\ \text{Rank(accuracy)} + w^{eff}\ \text{Rank(consistency)} + w^{const}\ \text{Rank(computational efficiency)}$$

where $w^{acc} = 0.4, w^{cont} = 0.5, w^{eff} = 0.1$. Due to missing or inaccurate information, computational efficiency was assigned a small weighting. Generally it should be weighted higher. Below we list the individual score rankings that contribute to the individual rank scores of the each of the three factors with the weightings we used given in parentheses. Note we choose everywhere to average over rank and not value to reconcile different scale ranges of scores and enable principled imputation of missing information using the average rank of remainder ranks.

1. *Accuracy* (w_{acc}=0.4)

   - Detection
     - class-specific mAP ($w_{\text{mAP}} = 0.35$)
     - class-specific IoU ($w_{\text{IoU}} = 0.3$)
     - confusion matrix score ($w_{\text{matrix}} = 0.35$)
   - Segmentation
     - class-specific s-score ($w_{\text{F}_2} = 1/3.$)
     - class-specific PPV ($w_{\text{PPV}} = 1/3.$)
     - class-specific recall ($w_{\text{Recall}} = 1/3.$)

2. *Consistency* (w^{const}=0.5)

   - Detection
     - (std./mean) class-specific mAP ratio ($w_{\sigma_{\text{mAP}}} = 1/3.$)
     - (std./mean) class-specific IoU ratio ($w_{\sigma_{\text{IoU}}} = 1/3.$)
     - class-specific generalization score$_{\text{g}}$ ($w_{\sigma_{\text{gen}}} = 1/3.$)
   - Segmentation
     - (std./mean) class-specific s-score ratio ($w_{\sigma_{\text{F}_2}} = 1/3.$)
     - (std./mean) class-specific PPV ratio ($w_{\sigma_{\text{PPV}}} = 1/3.$)
     - (std./mean) class-specific recall ratio ($w_{\sigma_{\text{Recall}}} = 1/3.$)

3. *Computational Efficiency* (w^{eff}=0.1)

   - □ Detection
     - Test time (s) rank
   - Architecture (network complexity)
   - Use of multiple networks (computational memory)
   - GPU rank

Individual classes were weighted artefact: 0.25, blur: 0.05, bubbles: 0.25, contrast: 0.05, instrument: 0.1, saturation: 0.1, specularity: 0.2 respectively. The weights are chosen to reflect the relative difficulty of the artefact class to restore (artefact and bubbles) and clinical importance (instrument). For computational efficiency, weights were assigned backbone or architecture: 0.3, use of multiple networks: 0.2, GPU: 0.1 and test speed: 0.4. For segmentation where test speed was not available for all methods, weights were assigned backbone or architecture: 0.5, use of multiple networks: 0.3, GPU: 0.2.

Supplementary Note III: Deep Neural Network Detection and Segmentation

We briefly review deep neural network approaches to detection and segmentation and propose a taxonomy based on algorithm design. The reader is referred to specialised reviews for technical details of detection[13] and segmentation[14]. Recently deep neural networks have emerged as the automatic method of choice for state-of-the-art object detection and segmentation objects both in everyday image scenes[10,15] and in biomedical images[16–18]. Compared to traditional feature handcrafting approaches deep neural networks construct features automatically by learning to associate between two datasets given sufficient training pairs. Specifically, given a set of input images x and the corresponding desired output, y_{true} (bounding boxes for detection or masks for segmentation), deep neural networks specify a parametrised function, $f(x;\theta)$ given by weights, θ that minimise the dissimilarity or loss between the function output and input x, $y = f(x;\theta)$ and the desired output y_{true} (Suppl. Fig. 18a). The performance of a deep learning system thus depends on the architecture design of f to capture the task complexity, how well the loss function captures the desired objective to be learnt and the ability of the chosen optimizer to find the best global minima. To this end, modern state-of-the-art architectures can be considered in terms of a 'backbone' network that extracts generically informative image features that feeds into a 'head' network to produce task-specific predictions (Suppl. Fig. 18b-d). The 'backbone' network is usually a pre-trained network with weights trained previously on a very large dataset and a related task such as image classification in PASCAL VOC[10] or MS COCO Challenges[15] to take advantage of the hierarchical feature learning of neural networks. Early layers learn generic "primitive" image concepts such as colour and edges that are generically useful across networks[19,20]. This practice is commonly called transfer learning and is used to train on smaller datasets. The "head" network is typically a very small network specifically designed for a given problem (Suppl. Fig.18c,d) and is trained for each dataset starting from randomly initialized or pretrained weights. To date, numerous deep learning object detection and segmentation systems have been proposed, however, most of these design share three common underlying considerations: scale (exploiting the image context), accuracy and speed. Scale is the ability to capture local and global level spatial relationships between image pixels to capture the specific characteristics of objects of any shape and size. Accuracy is the ability to maximize true positive and minimize false positive detection and speed refers to the computational time required by a trained network to process a single image. Often there is a speed-accuracy compromise. Suppl. Fig. 18e,f places EAD participants' base solutions within a taxonomy constructed according to design principle and chronological order of development.

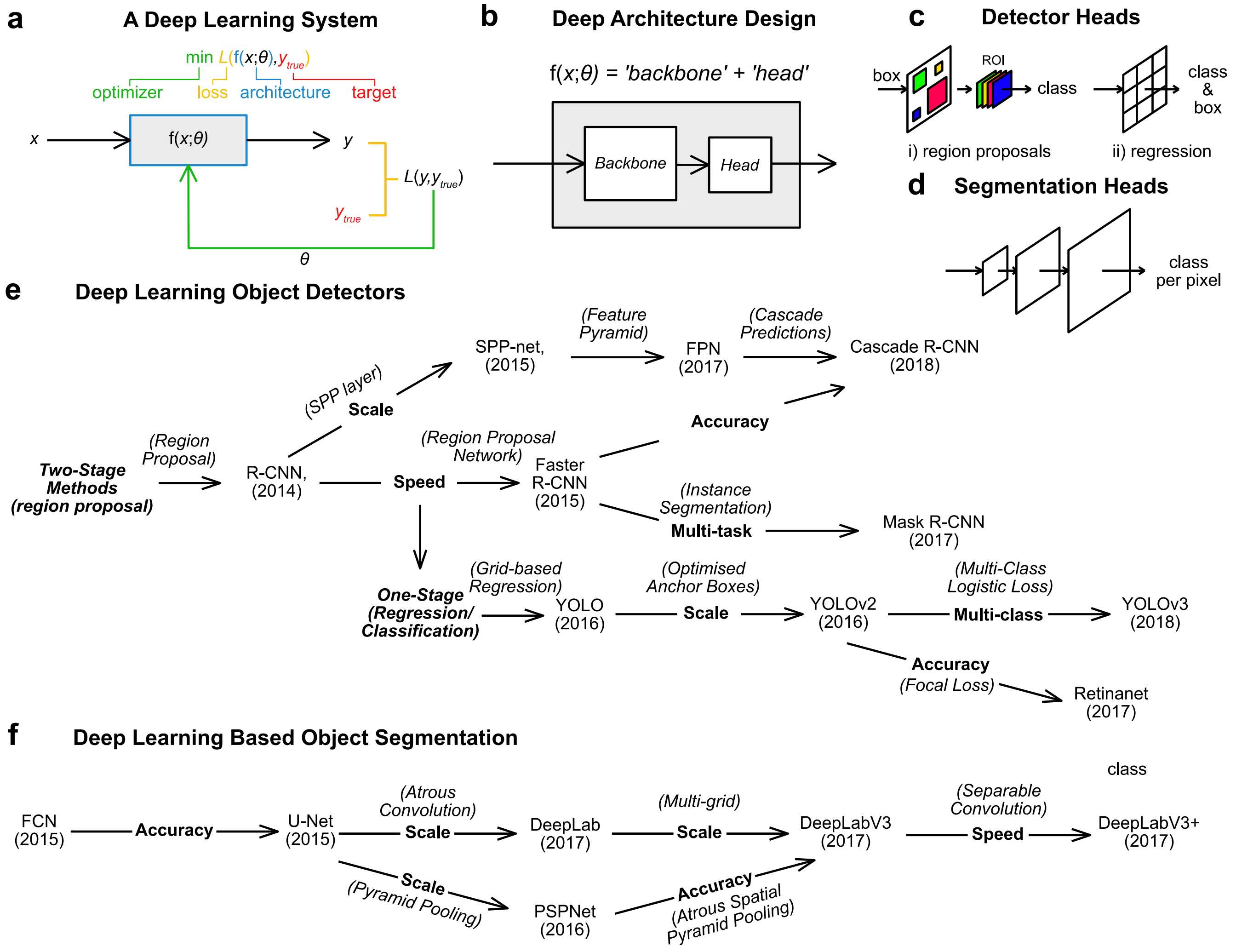

Figure 18. Architectural design of deep neural network object detectors and segmentation. **a**, Schematic of the design of a general deep neural network system. **b**, Schematic decomposition of a neural network into backbone and head sub-networks. The backbone network is constructed to extract image features through successive downsampling of the input image. Due to this property to 'encode' features, the backbone is also often termed an 'encoder'. Using the backbone 'encoded' features, the head network produces the task-specific output predictions. **c**, Two most common detector heads are: i) Region proposal classification – the backbone network predicts the coordinates of positive object containing candidate bounding box regions, the corresponding image patch is cropped out and classified into different object classes and background. ii) Regression-based classification – the joint backbone and head network acts as one large region proposal network that simultaneously predicts candidate bounding boxes and their class without intermediate proposal of candidate regions. **d**, Most common detector head found in medical imaging. The downsampled features from the backbone network is 'decoded' via parameterized upsampling to give a classification for each pixel at the original input image size. Combined with a backbone 'encoder', the full architecture is known as an 'encoder-decoder' network. **e, f**, Taxonomy of the submitted EAD2019 participant detection and segmentation algorithms, respectively. It is organized according to the architectural design principle (bold text between arrows, specific realization in brackets and italicized font) and in relation to historical development.

Supplementary Note IV: EAD Method summary

Below, we summarize the EAD2019 challenge methods for top 30% whose details are provided to us. Methods are detailed in the challenge proceeding[3].

yangsuhui. Team yangsuhui proposed to use Cascaded R-CNN with an additional layer of feature pyramid network (FPN) for endoscopic artefact detection. The multi-stage FPN was designed with an ResNet-101 backbone. The team used style transfer Cycle-GAN network to generate more training data for improved performance of the generalization task. For the segmentation task, the team used Deeplab-v3+ network with two different backbones ResNet-101 and MobileNet and 5 parallel convolution layers. All feature maps were merged and decoded to achieve the final segmentation.

zhangPY. An improved Mask R-CNN architecture with a flexible and multi-stage training protocol was used by team zhangPY. The team proposed a three step strategy: First, an instance segmentation was done using Mask R-CNN network on the semantic training datatset. Then, the trained Mask R-CNN model was subsequently used to predict instance masks for the training samples of detection task that have no pixel-level labels. During the inference process the results of object detection were replaced with the ground truth bounding boxes. Thus, predicted soft-level pixel segmentation were used at the third stage to train multiple Mask-aided R-CNN models with different backbone networks including ResNet-50, ResNet-101, FPN and their combinations. To obtain the final prediction map a graph clique-based approach was used for bounding box predictions.

Keisecker. Keisecker proposed an ensemble method using a one-stage RetinaNet detection network with ResNet backbones. The team pretrained ResNet-50 on MS COCO dataset, and ResNet-101 and ResNet-152 on ImageNet1k. The best performance for Keisecker was given by ensemble model with 7 different backbone architectures embedded with diverse data augmentations.

ilkayoksuz. ilkayoksuz proposed to use RetinaNet architecture with ResNet-152 backbone. The team built a multi-stage feature pyramid using ResNet-based feature pyramid network (ResNet-FPN). They generated a large derived dataset by augmenting the original images with free-form deformations to prevent over-fitting. To improve the speed, team ilkayoksuz decoded box predictions from only top 200 predictions per FPN level at confidence threshold of 0.36. They used a stratified 5-fold cross validation strategy to optimize the parameters of the network.

swtnb. swtnb combined two state-of-the-art deep object detection methods: YOLOv3 and Mask R-CNN. The team used Mask R-CNN with a feature pyramid network (FPN) and ResNet-101 backbone. The predictions from segmentation dataset were used to leverage the bounding box detection for the detection tasks. They additionally used a DNN model on patches with artefacts and performed data augmentation to avoid overfitting. Appropriate bounding boxes were achieved by performing using non maximum suppression.

akhanss. akhanss proposed to use optimized focal loss for dense object detection based on RetinaNet network pretrained with the ImageNet dataset. The team used ResNet-101 backbone. They applied several data augmentation and hyperparmeter tuning strategies. For semantic segmentation, team akhanss used an encoder-decoder U-Net model.

XiaokangWang. Faster R-CNN network with a FPN with ResNet-50 backbone was used by team XiaokangWang. The weights of their model were initialized with pre-trained weights on the COCO dataset. To capture the different sizes of artefacts, the team first obtained different size patches from the provided training dataset and then used scaling factors depending on the size of the object in the patch.

nqt52798669. Team nqt52798669 used Cascade R-CNN with ResNet-101 backbone for detection task. For semantic segmentation, they used Deep Layer Aggregation (DLA-60) model showing an improved performance over classical U-Net model.

ShufanYang. U-Net was used with ResNet-50 backbone pre-trained on ImageNet. The semantic segmentation training data was used for training for semantic segmentation and detection both.

[3] http://ceur-ws.org/Vol-2366/

References

1. Demšar, J. Statistical comparisons of classifiers over multiple data sets. *J. Mach. learning research* **7**, 1–30 (2006).
2. Dutta, A. & Zisserman, A. The VGG image annotator (VIA). Preprint at https://arxiv.org/abs/1904.10699 (2019).
3. Song, L. M. W. K. *et al.* Chromoendoscopy. *Gastrointest. endoscopy* **66**, 639–649 (2007).
4. Gono, K. *et al.* Appearance of enhanced tissue features in narrow-band endoscopic imaging. *J. biomedical optics* **9**, 568–578 (2004).
5. Singh, R., Mei, S. C. Y. & Sethi, S. Advanced endoscopic imaging in barrett's oesophagus: a review on current practice. *World journal gastroenterology: WJG* **17**, 4271 (2011).
6. Tanaka, S. *et al.* Pit pattern diagnosis for colorectal neoplasia using narrow band imaging magnification. *Dig. Endosc.* **18**, S52–S56 (2006).
7. He, K., Zhang, X., Ren, S. & Sun, J. Identity mappings in deep residual networks. In *European conference on computer vision*, 630–645 (2016).
8. Pech-Pacheco, J. L., Cristóbal, G., Chamorro-Martinez, J. & Fernández-Valdivia, J. Diatom autofocusing in brightfield microscopy: a comparative study. In *Proceedings 15th International Conference on Pattern Recognition*, vol. 3, 314–317 (2000).
9. Mittal, A., Moorthy, A. K. & Bovik, A. C. No-reference image quality assessment in the spatial domain. *IEEE Transactions on image processing* **21**, 4695–4708 (2012).
10. Everingham, M., Van Gool, L., Williams, C. K. I., Winn, J. & Zisserman, A. The PASCAL Visual Object Classes Challenge 2012 (VOC2012) Results. Online http://www.pascal-network.org/challenges/VOC/voc2012/workshop/index.html (2012).
11. Jonckheere, A. R. A distribution-free k-sample test against ordered alternatives. *Biometrika* **41**, 133–145 (1954).
12. Terpstra, T. J. The asymptotic normality and consistency of kendall's test against trend, when ties are present in one ranking. *Indagations Math.* **14**, 327–333 (1952).
13. Zhao, Z.-Q., Zheng, P., Xu, S.-t. & Wu, X. Object detection with deep learning: A review. *IEEE transactions on neural networks learning systems* 1–21 (2019).
14. Lateef, F. & Ruichek, Y. Survey on semantic segmentation using deep learning techniques. *Neurocomputing* **338**, 321–348 (2019).
15. Lin, T.-Y. *et al.* Microsoft COCO: Common objects in context. In *European conference on computer vision*, 740–755 (2014).
16. Yang, L., Zhang, Y., Chen, J., Zhang, S. & Chen, D. Z. Suggestive annotation: A deep active learning framework for biomedical image segmentation. In *Medical Image Computing and Computer Assisted Intervention*, 399–407, DOI: 10.1007/978-3-319-66179-7_46 (2017).
17. Ali, S. *et al.* A deep learning framework for quality assessment and restoration in video endoscopy. Preprint at http://arxiv.org/abs/1904.07073 (2019).
18. Urban, G. *et al.* Deep Learning Localizes and Identifies Polyps in Real Time With 96% Accuracy in Screening Colonoscopy. *Gastroenterology* **155**, 1069 – 1078, DOI: https://doi.org/10.1053/j.gastro.2018.06.037 (2018).
19. Zeiler, M. D. & Fergus, R. Visualizing and understanding convolutional networks. In *European conference on computer vision*, 818–833 (2014).
20. Hertel, L., Barth, E., Käster, T. & Martinetz, T. Deep convolutional neural networks as generic feature extractors. In *2015 International Joint Conference on Neural Networks*, 1–4 (2015).
